# Current structure predictors are not learning the physics of protein folding

**DOI:** 10.1093/bioinformatics/btab881

**Published:** 2022-01-31

**Authors:** Carlos Outeiral, Daniel A Nissley, Charlotte M Deane

**Affiliations:** Department of Statistics, University of Oxford, Oxford OX1 3PB, UK; Department of Statistics, University of Oxford, Oxford OX1 3PB, UK; Department of Statistics, University of Oxford, Oxford OX1 3PB, UK

## Abstract

**Summary:**

**Motivation.** Predicting the native state of a protein has long been considered a gateway problem for understanding protein folding. Recent advances in structural modeling driven by deep learning have achieved unprecedented success at predicting a protein’s crystal structure, but it is not clear if these models are learning the physics of how proteins dynamically fold into their equilibrium structure or are just accurate knowledge-based predictors of the final state.

**Results.** In this work, we compare the pathways generated by state-of-the-art protein structure prediction methods to experimental data about protein folding pathways. The methods considered were AlphaFold 2, RoseTTAFold, trRosetta, RaptorX, DMPfold, EVfold, SAINT2 and Rosetta. We find evidence that their simulated dynamics capture some information about the folding pathway, but their predictive ability is worse than a trivial classifier using sequence-agnostic features like chain length. The folding trajectories produced are also uncorrelated with experimental observables such as intermediate structures and the folding rate constant. These results suggest that recent advances in structure prediction do not yet provide an enhanced understanding of protein folding.

**Availability. **The data underlying this article are available in GitHub at https://github.com/oxpig/structure-vs-folding/

**Supplementary information:**

Supplementary data are available at *Bioinformatics* online.

## 1 Introduction

Protein folding, or how a protein attains its equilibrium 3D structure, is considered one of the grand challenges of modern molecular biology (Dill and MacCallum, 2012). If it were possible to accurately predict the folding pathway of a protein, it would have far-reaching implications for basic science, further the development of novel therapeutics and broaden the toolset for protein design and engineering. Some of the most prevalent aging-related pathologies, like Alzheimer’s (Selkoe and Hardy, 2016) or Parkinson’s disease (Kalia and Lang, 2015), originate when the delicate proteostasis machinery fails to ensure that proteins are correctly folded. The dynamical nature of the folding process also relates to other poorly understood phenomena like allostery (Campitelli *et al.*, 2020), fold-switching (Porter and Looger, 2018) or intrinsic disorder (Oldfield and Dunker, 2014). Even protein expression, one of the cornerstones of modern biotechnology, is highly dependent on folding: problems expressing recombinant proteins across different organisms are often attributed to changes in the folding mechanism due to different translation machinery (Mignon *et al.*, 2018). However, despite significant work (Dill and MacCallum, 2012; Outeiral *et al.*, 2021) we are still unable to accurately predict the folding pathway of a protein *de novo*.

Protein folding is often used as a misnomer for protein structure prediction, which is the prediction of the native state without regard to the pathway that the protein undergoes to attain it. The field of structure prediction has experienced significant progress over the past two decades, powered by the community-wide effort of the biennial CASP contest (Moult, 1996). This assessment exercise has witnessed multiple step changes in accuracy as novel ideas have been incorporated into the participant’s pipelines (Kryshtafovych *et al.*, 2014; 2019; Moult *et al.*, 2018). Although in earlier editions methods were inspired by the biophysical principles of folding, these were soon superseded by more successful knowledge-based approaches (Moult, 2005). In recent years, deep learning approaches have dramatically improved the quality of structure prediction. The introduction of deep learning techniques into protein structure prediction methods raised the average free modeling GDT_TS score, which measures structural similarity on a scale from 0 to 100, from 52.9 in CASP12 (Moult *et al.*, 2018), to 65.7 in CASP13 (Kryshtafovych *et al.*, 2019). In CASP14, a deep learning model, AlphaFold 2, achieved an average GDT_TS of 85.1 (Jumper *et al.*, 2021a). This method, and other similar techniques (Baek *et al.*, 2021), have been hailed as an acceptable solution to the protein structure prediction problem (Jumper *et al.*, 2021b).

These dramatic advances raise the question of whether these methods have achieved better understanding of protein folding physics, or are just successful at leveraging statistical knowledge of crystal structures into a prediction. To the best of our knowledge, the ability of structure predictors to model folding pathways has not been evaluated previously. Related work has studied the search trajectories of fragment replacement methods (Kandathil *et al.*, 2016), or attempted to introduce biological constraints into folding (de Oliveira *et al.*, 2018). Furthermore, recent work has shown that some deep learning predictors can pinpoint flexible residues (Schwarz *et al.*, 2020) or conformational changes (Del Alamo *et al.*, 2021), suggesting that these methods may capture dynamic phenomena reflected in the multiple sequence alignment. In this work, we examine whether protein structure prediction methods are able to reveal anything about a protein’s folding pathway.

We show that current protein structure prediction methods do not produce correct folding pathways. We first demonstrate that generated pathways have a weak link to formal folding kinetics, achieving a modest accuracy in discerning between protein chains that fold in a two-state or multistate mechanism. However, a simple sequence-agnostic feature, the length of the protein chain, is a far better predictor of folding dynamics. In the case of two-state folders, we also find that the dynamic trajectory is inconsistent with experimental folding rate constants. Finally, we demonstrate that predicted pathways produce erratic intermediates that are inconsistent with available hydrogen–deuterium exchange (HDX) data. We observe that most of the structure prediction methods are not significantly better than an unbiased coin and some of them are consistently worse at reproducing experimental measurements.

## 2 Materials and methods

### 2.1 Reference data

We compiled a dataset of 170 proteins for which experimental folding kinetics data is available. To produce this dataset, we collated entries from the Protein Folding Database (PFDB) of kinetic constants (Manavalan *et al.*, 2019) and the Start2Fold directory of HDX experiments (Pancsa *et al.*, 2016). We checked the annotations contained in the PFDB and changed the classification for human ubiquitin (PDB: 1UBQ) from multistate to two-state, given that the PFDB citation corresponds to a mutated species and the wild-type protein displays two-state kinetics (Jackson, 2006). The entries in the Start2Fold database do not include annotation for formal kinetics, so we manually annotated the results by querying the literature. The complete dataset and original publications are provided in Supplementary Table S1. We also compiled folding rate constants for a fraction of the proteins in this dataset that exhibit two-state kinetics, which are reported in Supplementary Table S2.

We collected available HDX data from Start2Fold and original papers (see Supplementary Data), to use as structural insight into the folding pathway (Clarke and Fersht, 1996). We observed that the residue-level annotation in the original database was sparse; we therefore queried the original sources and reconstructed the annotation as indicated in Supplementary Data. Each secondary structure element was labeled as structured or unstructured for each of the identified intermediates, on the basis of the experimental protection factors of the probes (in NMR experiments) or peptides (in mass spectrometry experiments) corresponding to a given portion of secondary structure.

Sequences and reference structures were downloaded from the RCSB PDB (Berman *et al.*, 2000) and trimmed according to the specifications of the entries. We used the codes referenced in the publications, even when higher resolution structures were available in the PDB. When using NMR structures with multiple models, the structure with the highest score was selected. Missing regions were repaired using MODELER (Webb and Sali, 2016) with standard parameters.

### 2.2 Trajectory generation

We generated protein folding trajectories using the latest versions, as of December 2020, of Rosetta (Schaap *et al.*, 2001), trRosetta (Yang *et al.*, 2020), DMPfold (Greener *et al.*, 2019), EVcouplings (Hopf *et al.*, 2019), RaptorX (Källberg *et al.*, 2012), SAINT2 (de Oliveira *et al.*, 2018) and the recently published RoseTTAFold (Baek *et al.*, 2021). We modified the source codes of the seven programs to print the current structure after every fragment substitution (for Rosetta and SAINT2); or after every 10 gradient updates (for trRosetta, RaptorX, DMPfold and EVfold, which use L-BFGS or related gradient descent algorithms); or after every refinement cycle in a SE(3)-equivariant iterative transformer (for RoseTTAFold). Given the large amount of data produced by Rosetta, averaging more than 200 000 snapshots per decoy, we subsampled the trajectories produced at every 100 fragment substitutions.

We preprocessed the sequences of our 170 test case proteins using the default pipelines provided by each piece of software, and used default parameters throughout. The generated trajectories for each of the 170 annotated proteins were compressed to the binary DCD format (Phillips *et al.*, 2005) and analyzed using in-house scripts. For RoseTTAFold, which produces only the atoms involved in the peptide bond, we used PULCHRA (Rotkiewicz and Skolnick, 2008) to reconstruct the β-carbons which are used in subsequent analysis. All information necessary to reproduce this study, including the diff files of the original source code, is available from https://github.com/oxpig/structure-vs-folding/.

We also considered trajectories generated by AlphaFold 2 (Jumper *et al.*, 2021b). Due to the architecture of the model, producing a trajectory would require training a replica of the AlphaFold Structure Module for every individual Evoformer iteration; this was done by Jumper *et al.* in the original publication, although the models have not been open-sourced. Fortunately, individual folding trajectories for each of the 170 proteins in our dataset were kindly provided by the DeepMind team. These trajectories were generated with the same methods and models as in the original publication (Jumper *et al.*, 2021b), save for the removal of any templates (although, of course, many of the structures were present in the training set).

### 2.3 Trajectory analysis

We analyzed the trajectories using the fraction of native contacts between secondary structure elements (Best *et al.*, 2013). These elements were identified using STRIDE (Frishman and Argos, 1995) on the crystal structure, ignoring any element shorter than four amino acids. Distances were calculated using MDAnalysis (Gowers *et al.*, 2019; Michaud-Agrawal *et al.*, 2011), and two amino acids were defined to be in contact if their β-carbons (α-carbons in the case of glycine) were less than 8.0 Å apart in the native structure. To account for fluctuations, we introduced a flexibility parameter ξ=1.2 whereby amino acids in contact in the crystal structure were still considered to be in contact in the simulated trajectory if their distance was *ξ* times the crystal structure distance. These parameter choices were inspired by the standard in the molecular dynamics literature (e.g. Nissley and O’Brien, 2018). To ensure that our conclusions were independent of the choice of parameters, we performed a parameter exploration on a reduced subset of the data (10 trajectories per protein)—see Supplementary Figure S1. This analysis is inspired by theoretical frameworks suggesting that many proteins fold by first forming secondary structure and then developing tertiary contacts between them (Englander and Mayne, 2017; Kim and Baldwin, 1982, 1990).

**Fig. 1.**
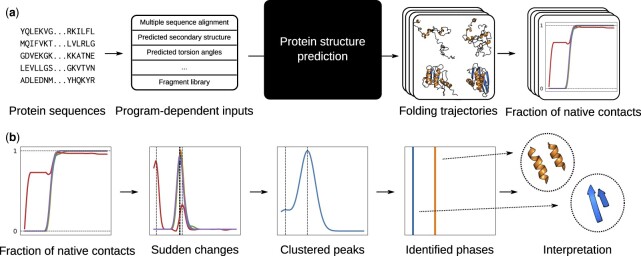
Proto col for the analysis of simulated folding pathways. (**a**) Trajectory generation process. Protein sequences are used to generate the necessary input features for a modified protein structure predictor using default processing scripts. The structure prediction software outputs detailed search trajectories, that are then summarized as the fraction of native contacts between pairs of secondary structure elements. (**b**) The trajectories are smoothed, and the positions of maximum change are identified via numerical differentiation. These peaks are subsequently clustered using KDE with a Gaussian kernel, allowing us to identify main phases of folding, and establishing whether the trajectory proceeds in one or more steps; and into the structural intermediates, which can be compared with HDX experiments

We computed the numerical time derivatives of the fraction of native contacts using finite differences and smoothed them using Friedman’s supersmoother (Friedman and Silverman, 1989) as implemented in the R stats package (R Core Team, 2013). The maximum value of the derivative for a pair of secondary structure elements was identified as the time point where the two of them are folded. We then fitted the data using a Gaussian Kernel Density Estimation (KDE) with bandwidth determined by Scott’s rule via SciPy (Virtanen *et al.*, 2020). When all of the folding transitions belong to a single peak, the trajectory was considered to be folding in two-states; when two or more peaks were found, the trajectory was labeled as multistate. Given the variability of the trajectories between prediction runs, many proteins had both two-state and multistate trajectories; hence we defined the fraction of two-state trajectories as the probability that a protein exhibits two-state kinetics. The trajectory generation and analysis process is reported in Figure 1.

## 3 Results

### 3.1 Pathways from protein structure predictors are worse than chain length at predicting formal kinetics

We first evaluated whether the predicted pathways from protein structure prediction methods are consistent with experimental refolding kinetics. The methods were asked to classify if a protein chain folds through two-state kinetics or multistate kinetics; in other words, whether the folding reaction is fully concerted or progresses through an intermediate. The ground truth is a dataset of *in vitro* refolding experiments extracted from the literature.

As described in Section 2, we modified the latest versions of seven state-of-the-art protein structure prediction methods to output their search trajectory. The first group, Rosetta and SAINT2, make use of a Monte Carlo minimization strategy based on fragment replacement. The second group, trRosetta, RaptorX, DMPfold and EVfold, use a flexible model with a simplified energy function as provided by CNS (Brünger *et al.*, 1998) or the Rosetta energy function (Alford *et al.*, 2017), in combination with inter-residue restraints derived from co-evolutionary data. Of these, one model (EVfold) uses binary contacts predicted by a Potts model (Hopf *et al.*, 2012), while the other three use deep learning to predict inter-residue distances (DMPfold) and possibly inter-residue orientations (trRosetta, RaptorX). The last method, RoseTTAFold, uses an iterative SE(3)-equivariant transformer that predicts protein structures in an end-to-end fashion without explicit minimization. These methods were used to produce 200 folding trajectories for each of the 170 proteins in our test set; except for the fragment replacement methods, SAINT2 and Rosetta, where due to high computational cost we generated only 10 trajectories per protein. This choice is justified, since these methods are known to present biases in their conformational search that lead to significant redundancy between independent trajectories (Kandathil *et al.*, 2016).

Generated pathways are influenced by the choices of the different protein structure prediction programs. Fragment replacement codes like SAINT2 and Rosetta start from the fully extended protein and slowly form compact states. Others like trRosetta and RaptorX start from a random conformation whose torsion angles have been selected from uniform sampling from a list of common torsion angles. RoseTTAFold initiates the trajectory in a compact structure that has been generated by inference on the MSA (and that often exhibits significant steric clashes). Despite the different initial states, all codes generate trajectories exhibiting complex folding dynamics.

The pathways were analyzed using a method based on the fraction of native contacts between secondary structure elements. In a concerted, two-state mechanism, we expect a sudden change where most of the interactions between the secondary structure elements of a protein form in a single step, while in a multistate mechanisms, we expect several sets of interactions forming at disjoint points of the trajectory. Our analysis (see Section 2) identifies the steepest changes, and uses a statistical criterion to determine whether they should be considered as a single group (two-state) or multiple groups (multistate, where the interleading peaks can be regarded as intermediates). Table 1 shows the results of this classification.

**Table 1.** Performance of the different protein structure prediction methods at determining folding kinetics

	RoseTTAFold	trRosetta	RaptorX	DMPfold	EVfold	SAINT2	Rosetta	Length
*10 Decoys*								
Unsupervised accuracy	0.614	0.614	0.560	0.565	0.552	0.554	0.552	—
Unsupervised F1-score	0.637	0.588	0.472	0.679	0.525	0.586	0.513	—
Supervised accuracy	0.607	0.576	0.551	0.588	0.568	0.538	0.527	**0.656**
Supervised F1-score	0.637	0.620	0.558	0.667	0.643	0.620	0.655	**0.731**
AUROC	0.675	0.654	0.626	0.594	0.605	0.608	0.560	**0.739**
*200 Decoys*								
Unsupervised accuracy	0.623	0.546	0.576	0.556	0.608	—	—	—
Unsupervised F1-score	0.663	0.638	0.610	0.687	0.616	—	—	—
Supervised accuracy	0.612	0.573	0.563	0.581	0.610	—	—	**0.656**
Supervised F1-score	0.649	0.640	0.565	0.667	0.645	—	—	**0.731**
AUROC	0.669	0.631	0.602	0.622	0.658	—	—	**0.739**

*Note*: Unsupervised metrics use a simple rule c(x) that assigns a protein the most frequent kinetics, i.e. if 50% or more of the decoys display multistate kinetics, the protein is taken to fold in multiple steps; otherwise it is considered two-state. Supervised metrics fit a logistic regression on c(x) and report the average of 1000 fivefold cross-validation experiments; note that the supervised score may sometimes be worse than the unsupervised one if the model does not generalize well. The baseline is a logistic regression that uses only the length of the protein. Accuracy reports the average recall per class, to account for the slight imbalance of the dataset (90 two-state folders and 80 multistate folders). The F1-score is the harmonic mean of recall and precision. The area under the receiver-operating curve (AUROC) for length is computed by projecting the values to the [0,1] interval. Bold indicates the top metric. We observe that chain length outperforms any of the protein structure prediction methods at predicting folding kinetics.

Prediction accuracies are modest, but significant. Using a bootstrap test (*N *=* *100 000), we determined that all the structure predictors are significantly superior to a random classifier (AUROC=0.500) at the 99% level of confidence. A randomized permutation test, however reveals that none of the predictors is significantly better at predicting folding kinetics than a linear classifier using only chain length. The fact that this sequence-agnostic classifier is better than any of the structure predictors suggests that, while protein structure prediction programs are capturing a non-trivial signal about folding, this signal is very weak.

The best predictor of folding kinetics appears to be RoseTTAFold (a deep learning model based on a transformer architecture which directly produces a structure from a multiple sequence alignment), closely followed by EVfold (based on energy minimization subject to evolutionary constraints). EVfold could be considered the most physically realistic method of those tested, since it does not modify the energy function to bias it toward the predicted native state. DMPfold is similar to EVfold, as it uses the same simulation engine (CNS), but the former uses a different method for introducing distance restraints: in DMPfold they are predicted with deep learning, whereas EVfold uses a Potts model. EVfold is a better predictor of folding kinetics than DMPfold, and also comparable to or better than RaptorX and trRosetta, which rely on deep learning. This suggests that, with the exception of RoseTTAFold, which belongs to a novel family of methods with physical assumptions baked into the model’s architecture, deep learning models are performing worse.

We also tested AlphaFold 2’s ability to predict folding kinetics, although in this case we had only one trajectory per protein. Using the method by Jumper *et al.* (2021b), we achieved an unsupervised accuracy of 0.613 and an unsupervised F1-score of 0.591 (note that other metrics, such as supervised scores or AUROC, are redundant, since the score is binary due to the availability of only one trajectory per protein), which may hint at a similar performance to RoseTTAFold. If after averaging over multiple decoys the performance metrics remained constant then this would reinforce the notion that deep learning methods based on SE(3)-equivariance might be capturing folding information encoded in the multiple sequence alignment.

Overall the quality of the structure prediction output does not appear to relate to the ability of the method to classify folding kinetics (see Supplementary Fig. S2). In the 10 decoy dataset there is a tendency toward the methods that generate worse structure predictions also being worse at predicting kinetics, but this effect may be a product of reduced sampling. If we consider the 200 decoy dataset the method that has the lowest structure prediction accuracy, EVfold, is the second best predictor of kinetics. Similarly for a given program, the quality of the predictions is largely independent of model quality (see Supplementary Fig. S2).

We examined one of the methods that use deep learning, DMPfold, in more detail. DMPfold uses an iterative process where prior predictions are used to refine the potential used in subsequent cycles. We compared the predictive power of multiple iterations, and observed that, while the area under the receiver-operating curve (AUROC) increases slightly with successive iterations, the overall accuracy is reduced (see Supplementary Fig. S3). The AUROC can be interpreted as the probability that a uniformly drawn two-state folder exhibits a higher proportion of two-state folding trajectories than a uniformly drawn multistate folder. This result suggests that, by iteratively refining predicted distances, the potential eliminates spurious predictions that might be a source of intermediates, as well as improve the final structure. However, since the accuracy is reduced, the description of the free energy hypersurface is not improved.

Finally, we found that some programs have an intrinsic bias toward predicting one or other folding mechanism. For example, for the majority of proteins, about 90% of the 200 DMPfold decoys exhibit two-state folding (hence the increase in AUROC from the 10 decoys sample to the 200 decoys sample), while RaptorX and EVfold tend toward predicting intermediates, and trRosetta presents a clear, but less marked bias toward two-state trajectories. These tendencies may explain the differences between unsupervised and supervised accuracy in Table 1.

Overall, these results suggest that protein structure prediction programs are not learning information about the folding mechanism.

### 3.2 Pathways from most protein structure predictors are uncorrelated with the rate constants of two-state folding

We next examined whether the protein structure prediction methods can predict the folding rate constant of the two-state processes. Our work follows that of Plaxco, Simons and Baker (Plaxco *et al.*, 1998), who demonstrated that the average contact order of the native structure is strongly correlated with the folding rate constant of two-state proteins. Follow-up papers have suggested that other measures, such as fractions of secondary structure (Gong *et al.*, 2003) or even predicted contacts (Punta and Rost, 2005), show similar correlations. We hypothesize that, if the folding pathways produced by protein structure methods were representative of folding, they should exhibit a similar relation, where the presence of the folding event in the trajectory is highly correlated with the folding rate constant.

We tested whether we could predict the folding rate constants of 79 two-state folding proteins from the PFDB (Manavalan *et al.*, 2019) (see Supplementary Table S2 for the experimental ground truth data). For each protein, we discarded all of the decoy trajectories that exhibited an intermediate and selected only two-state examples. In these trajectories, we localized the frame where the folding event started, and correlated its relative position in the full trajectory with the natural logarithm of the folding rate constant. As a baseline, we also computed the correlation with the average contact order and the chain length. We found that chain length outperformed average contact order at predicting the folding rate constant, counter to previous work that stated that length was not a useful predictor (Plaxco *et al.*, 1998). This is potentially due to the use of different examples and increased dataset size (our dataset is six times the size of that in the original paper).

**Fig. 2.**
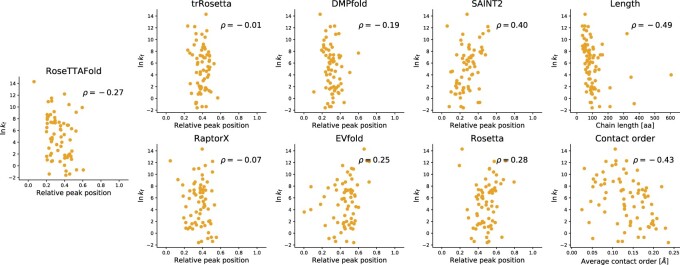
Correlation between the folding rate constant and folding events in simulated trajectories of the seven structure prediction methods considered, the length of the protein chain and the average contact order of the native structure. Every point represents the average over the maximum number of decoys possible (200 decoys for RoseTTAFold, trRosetta, RaptorX, DMPfold and EVfold; and 10 decoys for SAINT2 and Rosetta)

We found that most programs exhibit only a very weak correlation between the simulated trajectories and the folding rate constant (Fig. 2). The Spearman correlation coefficients are not significant, at the 95% level of confidence, for trRosetta and RaptorX and DMPfold, and while EVfold, RaptorX and Rosetta display significant correlation, the correlation has the wrong sign: later folding events lead to larger (faster) rate constants. In contrast, the correlation between trajectories produced by RoseTTAFold and folding kinetics, although weaker in magnitude, has the correct sign. Nevertheless, all of the methods are significantly worse than the length of the protein chain at predicting the folding rate constant.

We also found that AlphaFold 2 behaves similarly to RoseTTAFold, as found in the previous section. The Spearman correlation coefficient between the relative position of the folding event and the logarithm of the *k_f_* is –0.23, of the same order as RoseTTAFold and with the correct sign. Although the reduced number of decoys does not allow us to claim significance, the value suggests that the method is capturing some signal, and suggests that deep learning methods based on SE(3)-equivariance might detect the footprint that folding mechanisms have left in the multiple sequence alignment. However, it is unlikely that AlphaFold 2 would outperform the length of the protein chain at predicting the folding rate constant.

These results reinforce the conclusion that the ability of protein structure prediction methods to model folding pathways is inferior to trivial baselines.

### 3.3 Intermediates predicted by protein structure predictors are erratic and incompatible with available HDX data

As on occasion structure predictors do correctly identify folding kinetics, we next examine if in these cases, the structures predicted in the pathway are consistent with experimental data. We hypothesize that if the structure predictor has insight into the multistate process, it should (i) predict structures that are congruent with experimental measurements, and (ii) produce consistent predictions of the intermediates across independent replicas for the same protein. HDX experiments probe unfolded regions of a protein at different stages of the folding process and allow us to identify which regions of an intermediate are structured and which have not yet folded (see Supplementary Data for details). We compared the predicted folding trajectories to these data.

We use the predicted trajectories to identify which pairs of secondary structure elements are interacting closely in the intermediate. This allows comparison between the noisy protein structure prediction pathways and the low structural resolution provided by experimental HDX data. For every protein and program, we consider a binary vector whose elements correspond to pairs of secondary structure elements that are in contact in the native structure. We then use the same trajectory analysis as in the previous section to identify which pairs interact in the folding intermediate (or, in the case of fructose-biphosphate aldolase A, the first intermediate). The metrics of these classifiers are summarized in Table 2.

**Table 2.** Performance of the structure predictors at identifying the secondary structure interactions present in an intermediate

	RoseTTAFold	trRosetta	RaptorX	DMPfold	EVfold	SAINT2	Rosetta	Random
*200 Decoys*								
Accuracy	0.453	0.534	0.495	0.489	**0.540**	—	—	0.502
F1-score	0.222	0.169	0.110	0.026	**0.307**	—	—	0.252
Jaccard	0.052	0.052	0.052	0.052	0.052	—	—	**0.094**
AUROC	0.441	0.503	0.502	0.492	**0.530**	—	—	0.498

*Note*: The ground truth corresponds to a dataset of 11 proteins whose intermediates have been characterized with HDX experiments. Accuracy reports the average recall per class, to account for the slight imbalance of the dataset. The Jaccard score reflects the average Jaccard similarity of the predictions, expressed as a binary string (where 1 means that the native contacts between secondary structure elements are formed in the intermediate, while 0 means they are not), with the true answer. The random baseline corresponds to an unbiased coin predicting whether two secondary structure elements are in contact.

Intermediate structures are predicted with very low accuracy by all methods. A randomized permutation test shows that only one of the predictors, EVfold, exhibits predictive power superior to the random baseline. In contrast, RoseTTAFold is significantly worse than the random sample. This suggests that deep learning models are not learning the physics of folding, but rather collecting statistical information about crystal structures.

As an additional sanity check, we considered whether the structures generated throughout the trajectories are consistent with basic physical rules. We computed the clashscore (Davis *et al.*, 2004) of every snapshot in the first 10 decoys using Phenix (Adams *et al.*, 2010) and compared them against a threshold value of 30 clashes per 1000 atoms, determined as the 99th percentil of PDB structures with resolution ≤2.5 Å (see Supplementary Fig. S5). We observed that the majority of the methods produce a large number of structures with large clashes: methods based in CNS like DMPfold and EVfold produced over 80% of unphysical structures, and even the best methods like RaptorX and AlphaFold produced nearly 30–40% of structures with clashing atoms. This finding suggests that the potentials generated are not considering basic physical principles throughout the intermediate stages of the predictive process. This may explain the relative bad quality of intermediate predictions with respect to predictions of formal kinetics or the folding rate constant.

We then examined the variation between the predicted interactions by computing the Jaccard similarity between the binary vector of predicted interactions and the ground truth. This similarity is very low, in most cases worse than random, suggesting that independent replicas of the folding pathway by the protein structure prediction methods often lead to markedly different structural intermediates. These results once again imply that while the predictors may be good at modeling the energy hypersurface around the global minimum, they are not capturing other attractors and therefore produce erratic pathways.

The comparison with AlphaFold 2 suggests that the latter produces similar results. Of the nine proteins, seven are predicted with a Jaccard similarity of ≈0.1 to the ground truth (see Supplementary Fig. S6). The two proteins that are predicted with some accuracy, horse cytochrome C and cardiotoxin analogue III, are also the smallest in the dataset, which once again raises a concern of reduced entropic pressure. This suggests that AlphaFold 2 does not present any advantage at predicting the folding intermediates of a protein chain.

**Fig. 3.**
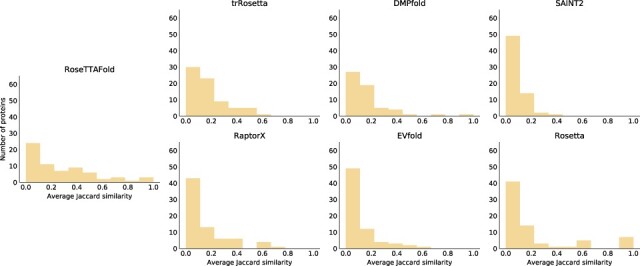
Average pairwise Jaccard similarity between multistate folding trajectories across all proteins in the dataset, for the seven structure prediction programs. Most methods exhibit significant variability between independent trajectories

We then investigated if these results extend from the proteins with HDX annotations, to the entire dataset of proteins we simulated. We computed the binary vectors for all pathways of multistate proteins exhibiting an intermediate, and computed the average Jaccard similarity for every protein (Fig. 3). The average pairwise Jaccard similarity is 0.1, and in most cases there are only a handful of proteins with an average over 0.5. The yeast cell-cycle control protein p13suc1 (PDB: 1PUC) is one of this handful; it presents only four native interactions, suggesting that this is again due to reduced entropic pressure. Overall, the pathways produced by protein structure prediction methods are erratic and generally inconsistent, suggesting that any ability to correctly predict multistate behavior does not arise from an understanding of the intermediates in the folding pathway.

## 4 Discussion

In this manuscript, we have investigated whether state-of-the-art protein structure prediction methods can provide any insight into protein folding pathways. We generated tens of thousands of folding trajectories with seven protein structure prediction programs (RoseTTAFold, trRosetta, RaptorX, DMPfold, EVfold, SAINT2 and Rosetta) and obtained a set of AlphaFold 2 trajectories, and used them to determine major features of folding using a simple set of statistical rules. We found that protein structure prediction methods can in some cases distinguish the folding kinetics (two-state versus multistate) of a chain better than a random baseline, but not significantly better, and often significantly worse, than a simple, sequence-agnostic linear classifier using only the number of amino acids in the chain.

Using a similar approach, we examined the relationship between simulated trajectories and other experimental observables: the folding rate constant of two-state folders, and the structure of intermediates in multistate trajectories. The simulated trajectories were in most cases not better than random at predicting the contacts formed in an intermediate, and in the case of predicting folding rate constants, none of the methods was superior to a linear classifier using the length of the protein chain.

Our results demonstrate that state-of-the-art protein structure prediction methods do not provide an enhanced understanding of the principles underpinning folding. Simulated trajectories from protein structure prediction methods are inconsistent with all available experimental data, in terms of folding mechanism, kinetics or structural data. In the general context of computational protein biophysics, our results suggests that current protein structure prediction programs, while now very successful at their primary role, are not an appropriate tool to investigate folding.

There are some limitations to our study. First of all, the concepts of folding intermediate and folding formal kinetics are imprecise. For example, many proteins have a tendency to form compact, molten globule structures, that may then fold cooperatively in a process that is referred to as ‘two-state’ (e.g. Di Paolo *et al.*, 2010). The folding mechanisms of multiple proteins have been widely discussed in the literature with conflicting results [e.g. for ubiquitin (Jackson, 2006) or T4 lysozyme (Kato *et al.*, 2007; Llinás *et al.*, 1999; Lu and Dahlquist, 1992)]. Folding is itself highly sensitive to an array of experimental conditions that includes temperature, pH and concentration of denaturant, and it may be difficult to discern when the methods are not correctly modeling the physics or simply portraying the wrong conditions.

While our results have shown the lack of consistency between the folding trajectories generated by protein structure prediction methods and experimental data, we have also seen that most structure predictors are better than random suggesting that a weak signal exists. The next stage will be to investigate how to extract the limited amount of folding information that is encoded in current protein structure prediction programs.

## Supplementary Material

btab881_Supplementary_Data
