# Supplementary material for: Current structure predictors are not learning the physics of protein folding
Source: Bioinformatics. 2022 Jan 31;38(7):1881–7. doi: 10.1093/bioinformatics/btab881 (PMC8963306; doi:10.1093/bioinformatics/btab881)
Supplement: btab881_Supplementary_Data [file btab881_supplementary_data.pdf]

Current structure predictors are not learning the physics of protein folding

Carlos Outeiral ,[1] Daniel A. Nissley ,[1] and Charlotte M. Deane [1]

[1] *Department of Statistics, University of Oxford, 24-29 St Giles', OX1 3PB, United Kingdom*

ABSTRACT

Predicting the native state of a protein has long been considered a gateway problem for understanding protein folding. Recent advances in structure prediction driven by deep learning have achieved unprecedented success at predicting a protein's crystal structure, but it is not clear if these models are learning the physics of how proteins dynamically fold into their equilibrium structure or are just accurate knowledge-based predictors of the final state. In this work, we compare the pathways generated by state-of-the-art protein structure prediction methods to experimental data about protein folding pathways. The methods considered were AlphaFold 2, RoseTTAFold, trRosetta, RaptorX, DMPfold, EVfold, SAINT2 and Rosetta. We find evidence that their simulated dynamics capture some information about the folding pathway, but their predictive ability is worse than a trivial classifier using sequence-agnostic features like chain length. The folding trajectories produced are also uncorrelated with experimental observables such as intermediate structures and the folding rate constant. These results suggest that recent advances in structure prediction do not yet provide an enhanced understanding of protein folding.

Keywords: protein folding, protein structure, coevolution, deep learning

1. SUPPLEMENTARY METHODS

1.1. *Coarse-grained molecular dynamics simulations*

Human ubiquitin (PDB: 1UBQ) is a small protein (76 amino acids) that has received significant attention in the protein folding literature. We performed molecular dynamics simulations for this protein to use as a baseline, using a native-centric coarse-grained force field where every amino acid is represented by a single bead centered on the α-carbon; for more detail, see Appendix 6. This formulation has been used to study protein folding in several previous studies *e.g.* (1; 2; 3). Our force field contains an adjustable scaling factor η which is determined by comparison to the experimental Gibbs free energy of folding. We ran replica exchange (REX) simulations and determined the ΔG of folding using the weighted-histogram analysis method (WHAM) (4), and found the parameter that reproduces the experimental $\Delta G_{\mathrm{folding}}$ (-7.11 kcal/mol) taken from the literature (5).

We produced temperature quenching simulations using a Langevin integrator (friction parameter 0.05 ps^{-1}, integration timestep 15 fs) and the OpenMM software package (6). We ran 200 independent trajectories consisting of 15 ns at 800 K, to induce temperature unfolding, followed by 300 ns at 298 K, to allow refolding. We printed trajectory snapshots every 5,000 timesteps (every 75 ps). The quenching trajectories were backmapped to a full backbone representation using PULCHRA (7), and analysed using the same procedure as the trajectories obtained from protein structure prediction methods.

1.2. *Intuition behind the analysis*

1.2.1. *Tertiary structure as contacts between secondary structure elements*

One of the assumptions of this study is that the formation of tertiary structure can be accurately monitored by examining the genesis of native contacts between pairs of secondary structure elements. This assumption is justified

Corresponding author: Charlotte M. Deane
deane@stats.ox.ac.uk

by the empirical observation that the protein structure prediction programs examined tend to form secondary structure very early in the trajectory, and then explore the assembly of these elements.

1. In fragment replacement methods, such as Rosetta and SAINT2, individual secondary structure elements are formed early due to the nature of the fragments. Fragment library generation strategies include predicted secondary structures (8; 9). This means that, after most residues have been part of a fragment substitution (which occurs in the early steps of the simulation), the protein will have acquired most of its secondary structure.

2. In methods that use potentials biased by predicted constraints (contacts, distances, interresidue angles, etc.) like trRosetta, RaptorX, DMPfold and EVfold, secondary structure forms early due to the existence of many constraints that are close in space (*e.g.* alpha-helices tend to form quickly due to the existence of many contacts between amino acids that are 4-6 residues apart).

3. In end-to-end deep learning methods, individual regions of the proteins tend to spontaneously form secondary structure at the beginning of the trajectory. Empirical examination of generated trajectories (*e.g.* the supplementary videos in (10)) suggests that secondary structure is formed in the first few iterations.

More broadly, this description also connects with theories of folding developed in the nineties, such as the "framework model" or the "diffusion-collision-adhesion" model that proposed, roughly, that proteins fold by first forming secondary structure and then developing tertiary contacts between them (11; 12).

1.2.2. *Intermediates and the maximum point of the derivative*

In this study, we use the heuristic that the maxima of the derivative of the smoothed fraction of native contacts can be used to identify the presence of intermediates. The physical intuition behind this heuristic is that, if a protein folds via a two-state mechanism, then all of its tertiary interactions (which, as explained above, can be safely equated with contacts between pairs of secondary structure elements) should form roughly at the same time. Therefore, it is possible to classify the folding mechanism by monitoring the time series of native contacts for every pair of secondary structure elements: if they all experience a sudden increase at a similar time, then it may be concluded that folding is roughly 2-state; however, if some pairs of secondary structure elements associate first, and then others some time later, then this points towards the existence of transient structures, and thus a multistate mechanism.

There exist many alternative heuristics which could be used to analyse this behaviour. One trivial approach would be to introduce a threshold θ, and determine that any change in the fraction of native contacts superior to θ implies a significant structural rearrangement. One may then verify whether all of the changes happen at once, or at the same time. In this work we decided to use an approach that does not require to introduce an arbitrary parameter and instead considers "jumps" in the time series.

2. CASE STUDY: UBIQUITIN

Ubiquitin is a 76-residue protein involved in signaling, notably by marking proteins for degradation by the 26S proteasome (13). The folding and dynamics of ubiquitin have been widely studied both experimentally (14) and computationally (15; 16), including by millisecond all-atom unbiased molecular dynamics simulations of folding (17). The kinetics of folding were an object of controversy in the late nineties, with claims of a three-state mechanism (18), although the consensus opinion in the literature is now that ubiquitin is a two-state folder (19; 20).

We analysed the protein structure prediction trajectories for ubiquitin generated during our analysis, and as a physically-inspired baseline we considered a coarse-grained molecular dynamics (CGMD) simulation. Representative trajectories for each program are provided in Supplementary Videos 1 to 9. We found that ubiquitin displays two-state folding kinetics in most of the simulated trajectories of RoseTTAFold, trRosetta, DMPfold and EVfold, but only in less than one quarter in RaptorX (see Figure 1b). Surprisingly, CGMD simulations also suggested that the folding is multistate, with 62% of the trajectories exhibiting an intermediate. These results suggest that all codes, except potentially RoseTTAFold and DMPfold, are generating stable intermediates that do not reflect experimental kinetics.

We then examined the structures of the intermediates, and found significant variability. Most programs, including the CGMD reference, have a tendency towards the β-strands A and B interacting (see Figure 2a), signaling the formation of a β-hairpin. This feature has been observed experimentally in NMR studies of unfolded ubiquitin (21) in up to 8M urea, and could suggest that the predictors are identifying strong interactions that govern the formation of metastable structures. However, most of the structure predictors also exhibit some spurious interactions that are

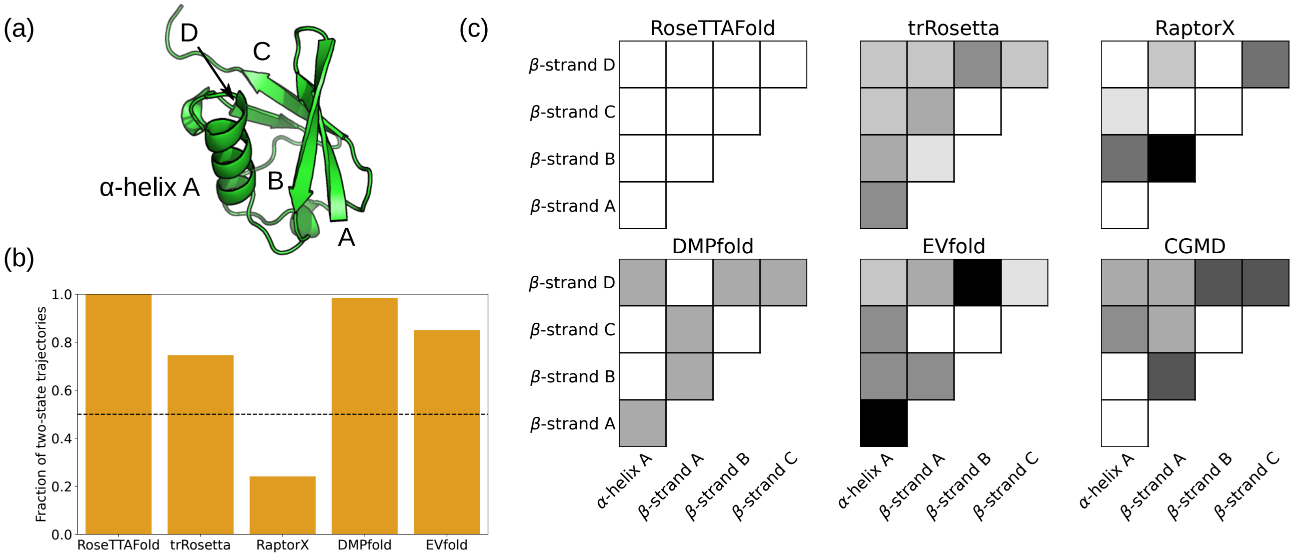

Figure 1. Simulations of human ubiquitin folding. (a) Native structure of human ubiquitin (PDB: 1UBQ). (b) Proportion of trajectories generated by each program that exhibit two-state dynamics. (c) Pairs of secondary structure elements that are formed in the identified intermediate. Color represents the proportion of trajectories where an intermediate presents a given interaction, where white is 0 (does not appear in any trajectory) and black is 1 (appears in all trajectories). Note that none of the RoseTTAFold trajectories exhibits an intermediate, hence none of the interactions is identified.

not observed in either the CGMD reference or the experimental data, such as interactions between the α-helix and the β-strands A and B (see Figure 2a). This suggests that, while the structure predictors do capture some interesting interactions, these are likely to be of limited use, since they are hidden within large amounts of spurious information.

We next inspected the simulated trajectories visually. As Figure 2 shows, despite RoseTTAFold exhibiting clear two-state dynamics according to the collective variables, all of its trajectories display unphysical behaviour. Initial structures are characterised by unrealistic steric clashes and violations of the geometry of the peptide bond, and are followed by a relaxation of the backbone into the final structure.

trRosetta and RaptorX present trajectories with comparable dynamics, probably due to the similarities between their protocols. In these trajectories, the protein exhibits random movements in the unfolded state, followed by collapse and formation of secondary structure, concomitant with the activation of the biasing potential, and finally small local exploration of the positions of the loops. This mechanism is consistent with the CGMD simulations, although in several snapshots the protein gets trapped in local minima of the biasing potential, which gives rise to detected intermediates.

DMPfold and EVfold, despite using a similar approach (the CNS optimisation engine in combination with predicted contacts), exhibit very different folding trajectories. DMPfold starts in a structure consistent with predicted distances, and explores the neighbourhood of this structure, whereas EVfold starts in an elongated state, experiences collapse, and then explores several potential conformations, with no apparent relation between them, until it finds the best structure according to its energy function. Both mechanisms are inconsistent with the CGMD simulations.

The AlphaFold 2 trajectory is similar to RoseTTAFold. The initial frames exhibit significant steric clashes, which are resolved after about 10 Evoformer iterations, and are followed by small oscillations around the equilibrium structure that are reminiscent of a protein subject to harmonic restraints. These results once again support the hypothesis that these methods are not learning the free energy hypersurface, but only the small free energy funnel that surrounds the native state.

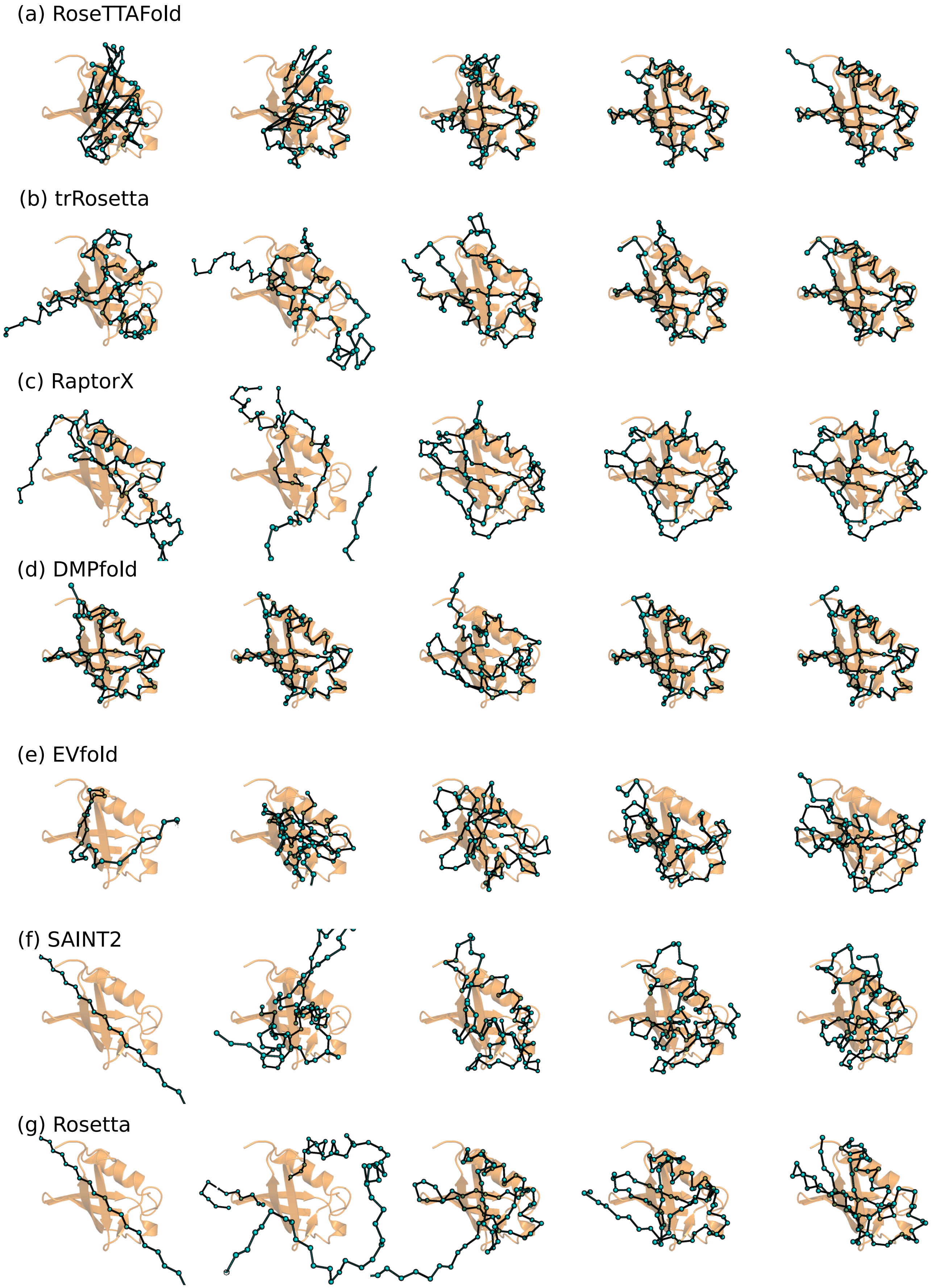

Figure 2. Representative snapshots of the simulated folding trajectories for human ubiquitin, for the seven structure prediction programs. The α-carbons are represented as blue beads joined by dark rods. Every structure has been aligned to the crystal structure (PDB: 1UBQ), which is shown as an orange cartoon in the background.

3. EXPERIMENTAL FOLDING KINETIC DATA

PDB code	Kinetics	Publication
1A64	Multistate	10.1021/bi9629283
1A6N	Multistate	10.1006/jmbi.1998.2273
1ADO	Multistate	10.1021/bi027388q
1ADW	Multistate	10.1006/jmbi.1998.2588
1AM7	Multistate	10.1021/bi101126f
1APS	Two-state	10.1110/ps.041205405
1ARR	Two-state	10.1021/bi961375t
1AU7	Multistate	10.1073/pnas.1101752108
1AUE	Multistate	10.1021/ja016480r
1AVZ	Two-state	10.1110/ps.041205405
1AYI	Multistate	10.1038/83074
1B9C	Multistate	10.1021/bi048733+
1BA5	Two-state	10.1073/pnas.1835776100
1BDD	Two-state	10.1002/pro.5560060709
1BE9	Multistate	10.1016/j.jmb.2004.11.040
1BKS	Multistate	10.1016/j.jmb.2005.01.064
1BNI	Multistate	10.1016/j.jmb.2003.08.024
1BTA	Multistate	10.1006/jmbi.1994.0196
1C8C	Two-state	10.1006/jmbi.2000.4234
1C9O	Two-state	10.1038/nsb0398-229
1CBI	Multistate	10.1002/(SICI)1097-0134(19981001)33:1<107::AID-PROT10>3.0.CO
1COE	Multistate	10.1016/j.abb.2006.01.003
1CSP	Two-state	10.1038/nsb0398-229
1CUN	Two-state	10.1016/j.jmb.2004.09.037
1D6O	Two-state	10.1110/ps.041205405
1DIV	Two-state	10.1110/ps.041205405
1DKT	Two-state	10.1016/s0022-2836(02)01202-0
1DWR	Multistate	10.1073/pnas.0305376101
1E0G	Two-state	10.1016/j.jmb.2008.05.020
1E0L	Two-state	10.1016/j.jmb.2006.05.050
1E0M	Two-state	10.1021/bi9822630
1E3Y	Two-state	10.1007/s00249-011-0756-6
1E+041	Two-state	10.1016/j.jmb.2009.04.004
1EHB	Two-state	10.1021/bi990550d
1EKG	Multistate	10.1038/srep20782
1ENH	Multistate	10.1073/pnas.1835776100
1F21	Multistate	10.1073/pnas.1305887110
1FA3	Multistate	10.1021/bi701142a
1FEX	Two-state	10.1073/pnas.1835776100
1FGA	Two-state	10.1074/jbc.274.48.34083
1FHT	Two-state	10.1110/ps.041205405
1FNF	Two-state	10.1006/jmbi.1997.1148

PDB code	Kinetics	Publication
1FTG	Multistate	10.1021/bi010216t
1G6P	Two-state	10.1038/nsb0398-229
1GM1	Two-state	10.1093/protein/gzi047
1GXT	Multistate	10.1016/s0022-2836(03)00627-2
1HCD	Two-state	10.1016/j.jmb.2004.09.091
1HCE	Two-state	10.1110/ps.31702
1HDN	Two-state	10.1021/bi9717946
1HEL	Two-state	10.1038/349633a0
1HFX	Multistate	10.1021/bi00072a025
1HFZ	Multistate	10.1006/jmbi.1999.2687
1HNG	Multistate	10.1021/bi971294c
1HRC	Multistate	10.1021/ja410437d
1HRH	Multistate	10.1002/pro.5560071014
1I1B	Multistate	10.1021/bi026197k
1IDY	Two-state	10.1073/pnas.1835776100
1IFC	Multistate	10.1021/bi012042l
1IGS	Multistate	10.1016/s0022-2836(02)00557-0
1IMQ	Two-state	10.1110/ps.041205405
1IO2	Two-state	10.1016/j.jmb.2008.02.039
1J5U	Two-state	10.1110/ps.041205405
1JO8	Two-state	10.1110/ps.041205405
1JOO	Multistate	10.1110/ps.28202
1K0S	Two-state	10.1110/ps.041205405
1K85	Two-state	10.1016/j.jmb.2007.09.088
1K8M	Two-state	10.1016/s0014-5793(02)03444-0
1K9Q	Two-state	10.1073/pnas.1008026107
1KDX	Two-state	10.1021/acschembio.7b00289
1L63	Multistate	10.1006/jmbi.1999.3204
1L8W	Two-state	10.1110/ps.041205405
1LMB	Two-state	10.1110/ps.041205405
1LOP	Two-state	10.1006/jmbi.2000.3580
1LZ1	Multistate	10.1021/bi00185a026
1M9S	Two-state	10.1110/ps.041205405
1MBC	Multistate	10.1073/pnas.0804033105
1MJC	Two-state	10.1002/pro.5560070228
1N88	Two-state	10.1110/ps.041205405
1NFI	Two-state	10.1016/j.jmb.2011.02.021
1NTI	Multistate	10.1073/pnas.152321499
1O6X	Two-state	10.1110/ps.041205405
1OKS	Multistate	10.1074/jbc.M116.721126
1OMP	Multistate	10.1073/pnas.1319482110
1ONC	Multistate	10.1021/bi900596j
1OPA	Multistate	10.1002/(sici)1097-0134(19981001)33:1<107::aid-prot10>3.0.co
1OSP	Multistate	10.1016/s0022-2836(02)00882-3
1PGB	Multistate	10.1038/13311
1PHP	Multistate	10.1021/bi961330s
1PIN	Two-state	10.1006/jmbi.2001.4873

PDB code	Kinetics	Publication
1PNJ	Two-state	10.1016/j.jmb.2010.08.046
1PRB	Two-state	10.1021/jp049652q
1PRS	Two-state	10.1016/s0014-5793(98)01287-3
1PUC	Multistate	10.1016/s0969-2126(00)00084-8
1QAU	Two-state	10.1016/j.febslet.2007.02.011
1QLP	Multistate	10.1016/j.jmb.2012.08.019
1QTU	Two-state	10.1006/jmbi.2001.4928
1R2T	Multistate	10.1016/j.jmb.2003.12.076
1RA9	Multistate	10.1016/s0022-2836(02)01444-4
1RBX	Multistate	10.1073/pnas.87.21.8197
1RFA	Two-state	10.1016/j.jmb.2006.10.079
1RG8	Two-state	10.1016/s0022-2836(03)00321-8
1RIS	Two-state	10.1110/ps.041205405
1RYK	Two-state	10.1110/ps.041205405
1SHG	Two-state	10.1110/ps.041205405
1SPR	Two-state	10.1110/ps.041205405
1SRL	Two-state	10.1110/ps.041205405
1SS1	Two-state	10.1016/j.jmb.2006.05.051
1ST7	Two-state	10.1002/prot.20340
1TEN	Two-state	10.1006/jmbi.2000.3517
1THF	Multistate	10.1021/bi300189f
1TIT	Multistate	10.1016/s0969-2126(01)00596-2
1TP3	Two-state	10.1073/pnas.0804774105
1TTG	Multistate	10.1110/ps.9.1.112
1U4Q	Two-state	10.1016/j.jmb.2004.09.037
1UBQ	Multistate	10.1021/cr040430y
1UCH	Multistate	10.1111/j.1742-4658.2009.06990.x
1UZC	Multistate	10.1073/pnas.0401732101
1V9E	Multistate	10.1016/j.bbrc.2008.02.096
1VII	Two-state	10.1016/s0022-2836(03)00519-9
1W4E	Two-state	10.1016/j.jmb.2005.12.016
1W4J	Two-state	10.1016/j.jmb.2008.06.081
1WIT	Two-state	10.1016/S0969-2126(99)80181-6
1WQ5	Multistate	10.1016/0022-2836(94)90023-x
1YGW	Multistate	10.1021/bi00075a006
1YMB	Multistate	10.1021/ac101679j
1YOB	Multistate	10.1021/ja8089476
1YYJ	Two-state	10.1021/bi025872n
1YYX	Two-state	10.1021/bi025872n
2A3D	Two-state	10.1073/pnas.2136623100
2A5E	Multistate	10.1006/jmbi.1998.2420
2ABD	Multistate	10.1006/jmbi.2000.4003
2BJD	Multistate	10.1021/bi030238a
2BKF	Two-state	10.1021/bi1016793
2CRO	Multistate	10.1021/bi001388d
2CRT	Multistate	10.1074/jbc.273.17.10181
2EQL	Multistate	10.1006/jmbi.1999.2741

PDB code	Kinetics	Publication
2FS6	Multistate	10.1002/prot.1040
2GA5	Two-state	10.1039/C3CP54055C
2J5A	Two-state	10.1016/j.jmb.2006.09.016
2JMC	Two-state	10.1093/protein/gzp041
2KDI	Multistate	10.1016/j.bpc.2011.05.004
2KLL	Multistate	10.1371/journal.pone.0144067
2L6R	Two-state	10.1021/ja801401a
2LLH	Two-state	10.1073/pnas.0910516107
2LZM	Multistate	10.1016/j.jmb.2006.10.048
2MYO	Two-state	10.1073/pnas.0604653104
2PQE	Multistate	10.1016/j.jmb.2003.07.002
2PTL	Two-state	10.1110/ps.041205405
2QJL	Two-state	10.1110/ps.041205405
2RN2	Multistate	10.1038/12277
2VH7	Two-state	10.1021/bi9822630
2VIL	Multistate	10.1006/jmbi.2000.4190
2VKN	Two-state	10.1110/ps.041205405
2WQG	Two-state	10.1016/j.febslet.2015.06.002
2WXC	Two-state	10.1016/j.jmb.2008.12.056
2X7Z	Two-state	10.1074/jbc.m110.110833
3BLM	Multistate	10.1016/0022-2836(85)90384-5
3CHY	Multistate	10.1021/bi00185a025
3CI2	Two-state	10.1110/ps.041205405
3F6R	Multistate	10.1016/j.jmb.2009.11.008
3H08	Multistate	10.1021/bi900305p
3NPO	Multistate	10.1006/jmbi.1999.3515
3O49	Two-state	10.1016/j.jmb.2011.02.002
3O4B	Two-state	10.1016/j.jmb.2011.02.002
3O4D	Two-state	10.1016/j.jmb.2011.02.002
3ZRT	Two-state	10.1016/j.febslet.2007.02.011
4BLM	Multistate	10.1021/bi0358162
5DFR	Multistate	10.1002/pro.5560040204
5L8I	Multistate	10.1002/prot.22286
9PCY	Multistate	10.1021/bi00097a005

Table S1. Experimental folding data used in this work

4. EXPERIMENTAL TWO-STATE FOLDING RATE CONSTANTS

PDB code	$\ln k_f$	PDB code	$\ln k_f$
1ARR	9.20	1BA5	5.90
1E41	6.90	1FEX	8.20
1IDY	8.70	1IMQ	7.33
1L8W	3.60	1LMB	10.40
1PRB	14.30	1RYK	9.10
1SS1	11.50	1ST7	10.90
1U4Q	11.00	1VII	12.30
1W4E	10.20	1W4J	12.30
1YYJ	8.40	2A3D	12.20
2LLH	7.90	2WXC	11.70
2WQG	8.80	1KDX	8.20
1IO2	-1.40	2MYO	4.80
2QJL	2.60	1APS	-1.60
1D6O	1.60	1DKT	5.80
1E0G	7.90	1FHT	4.60
1HDN	3.30	1J5U	6.90
1N88	2.00	1O6X	6.80
1RFA	7.70	1RIS	6.10
1SPR	8.70	2BKF	6.20
2J5A	7.30	2PTL	4.10
2VH7	0.72	3CI2	5.80
1EHB	4.50	2GA5	5.40
1NFI	1.80	1C8C	7.20
1K0S	7.40	1LOP	7.40
1C9O	7.20	1CSP	6.50
1G6P	6.30	1E0L	10.70
1E0M	8.90	1HCD	1.60
1JO8	2.50	1K85	1.40
1K8M	-0.71	1TP3	3.00
1K9Q	8.40	1M9S	4.00
1MJC	5.30	1PIN	9.20
1PNJ	-0.69	1QTU	0.08
1RG8	1.30	1AVZ	4.90
1SHG	1.10	1SRL	4.40
1TEN	1.80	1WIT	0.85
2VKN	2.10	1FNF	-0.90
1GM1	1.00	2JMC	3.30
1FGA	-1.40	1QAU	1.80
3O4D	4.90	2L6R	9.90
2X7Z	0.74		

Table S2. Folding rate constants

5. EXPERIMENTAL STRUCTURAL FOLDING DATA

5.1. *Fructose-bisphosphate aldolase A*

Pan and Smith (22) studied the folding of rabbit muscle aldolase (PDB: 1ADO) using HDX-MS. The authors proposed a folding mechanism whereby an initial collapsed state is formed cooperatively from the union of four widely separated regions of the backbone, followed by two sequential folding steps of individual domains. The authors annotated the regions of the protein as corresponding to each of these intermediates, by means of the peptides obtaining during HPLC-MS analysis.

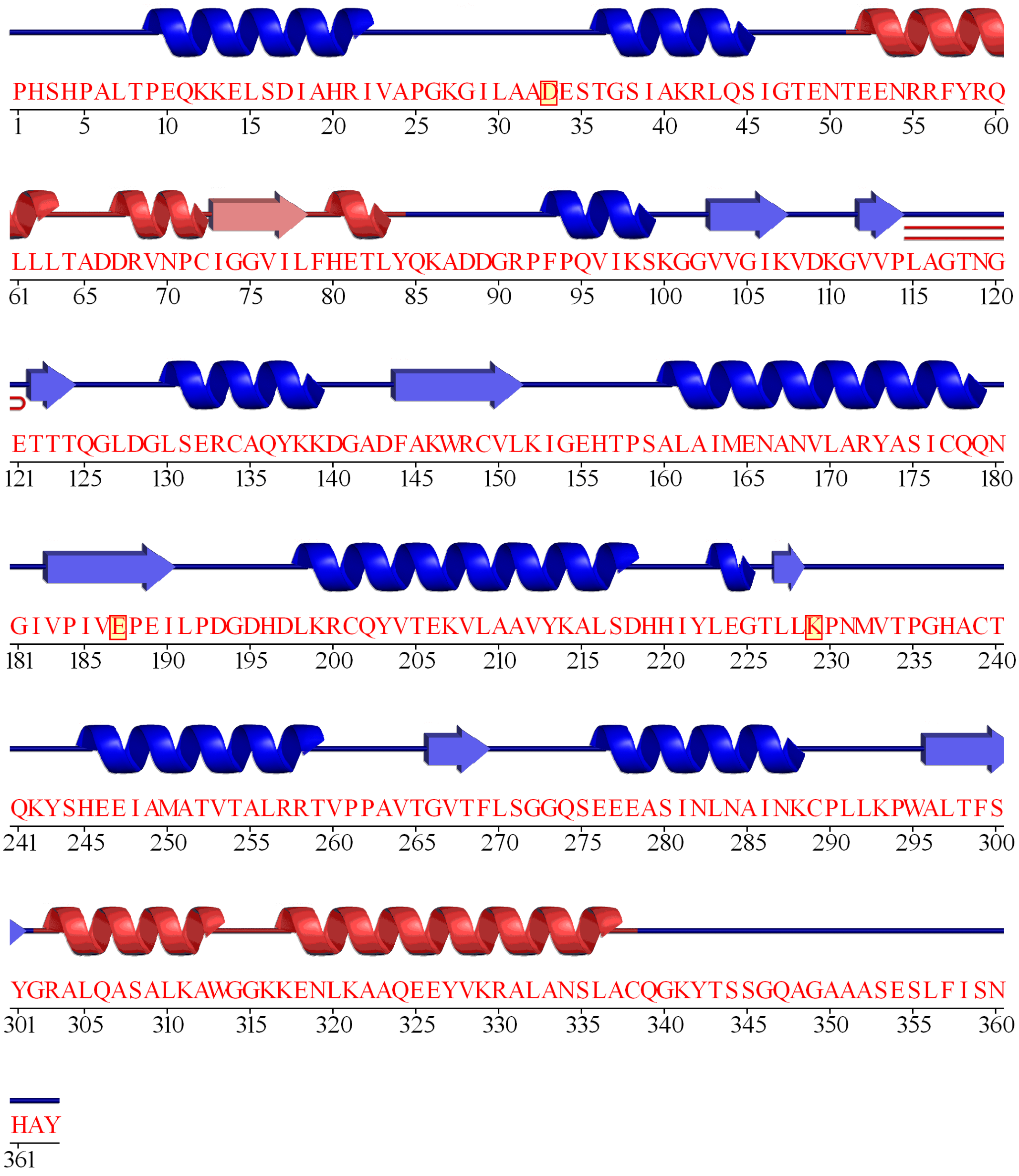

5.2. *Alpha-subunit of tryptophan synthase*

Wintrode et al. (23) studied the folding of the α-subunit of tryptophan synthase (PDB: 1BKS, for an E. coli homologous with 85% sequence identity), a TIM barrel protein from *E. coli*, using HDX-MS. The authors identify an obligate intermediate, which comprises most of the N-terminal region.

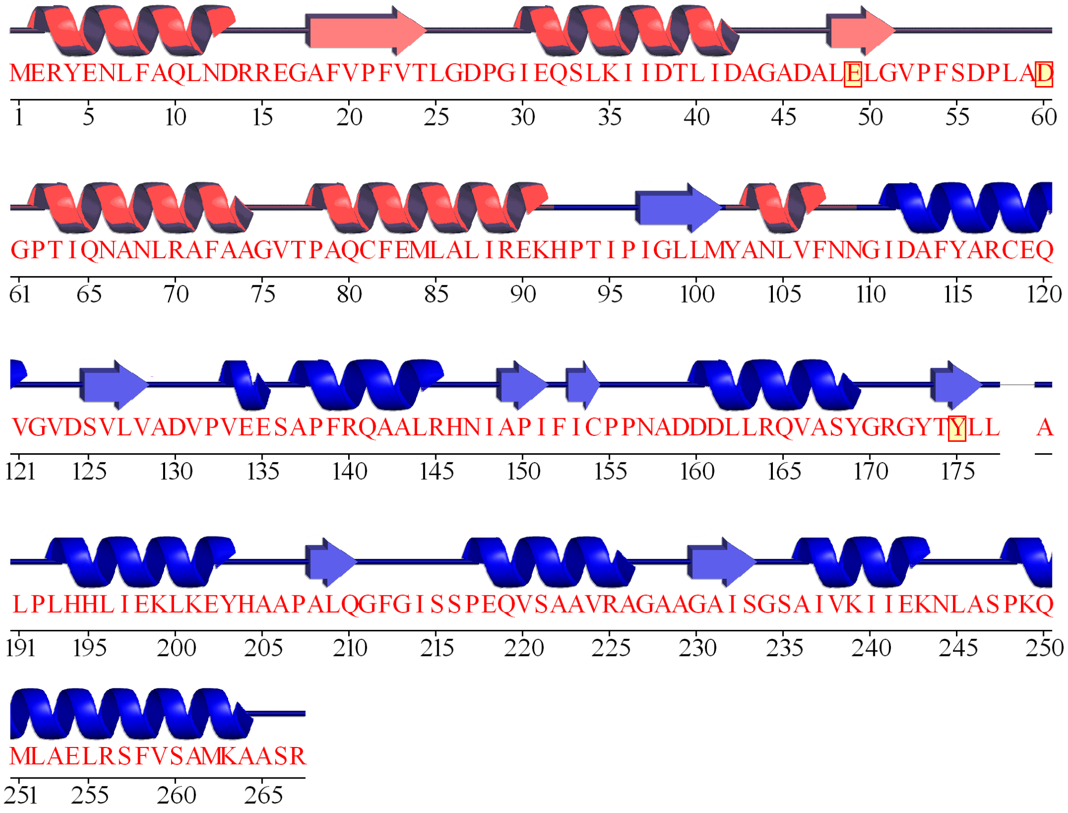

5.3. *Cytochrome C*

Roder et al. (24) studied the folding of horse's cytochrome C (PDB: 1HRC) using HDX-NMR. The authors observe that the contact between the C-terminal and N-terminal helices is formed early in folding, followed by structuring of the rest of the protein. A study by Elove et al. (25) identified multiple possible folding pathways originating from different coordinations to the heme group. A study by Fazelinia et al. (26) investigated folding during the first 140 μs using a microfluidics device and HDX-NMR, showing that interactions between α-helices drives condensation at the start of folding.

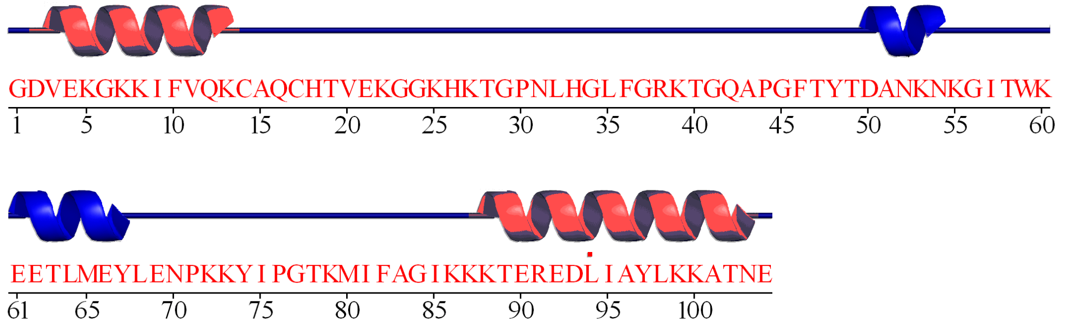

5.4. *Staphilococcal nuclease H124L*

Walkenhorst et al. (27) studied the folding of staphilococcal nuclease with a H124L mutation (PDB: 1JOO). Their data shows that the formation of the β-barrel domain, in particular the β-hairpin formed by strands 2 and 3 and a site in the C-terminus, precedes the formation of the α-helical domain.

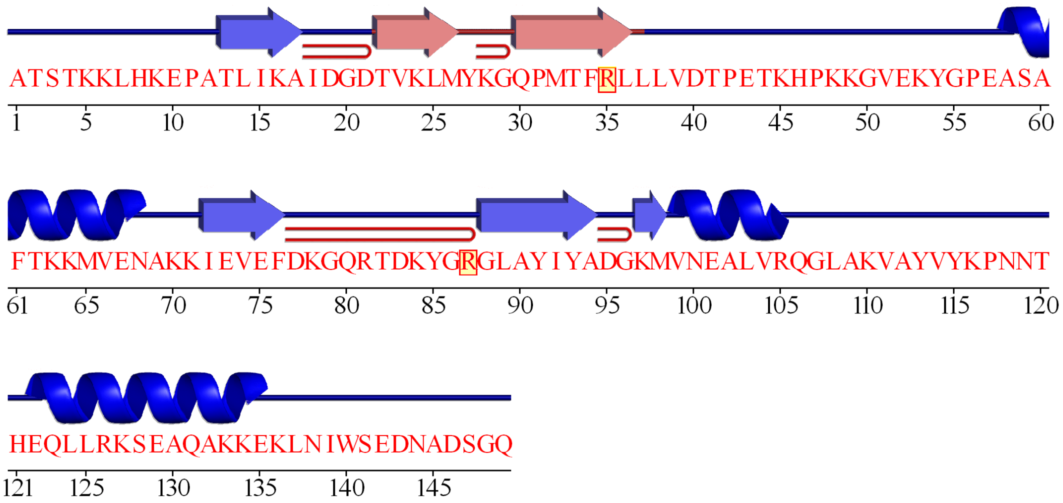

5.5. *Triosephosphate isomerase*

Pan et al. (28) studied the folding of rabbit triosephosphate isomerase (PDB: 1R2T) using HDX-MS. They found that the C-terminal half folds to form the intermediate, which then forms a TIM barrel with the N-terminal half.

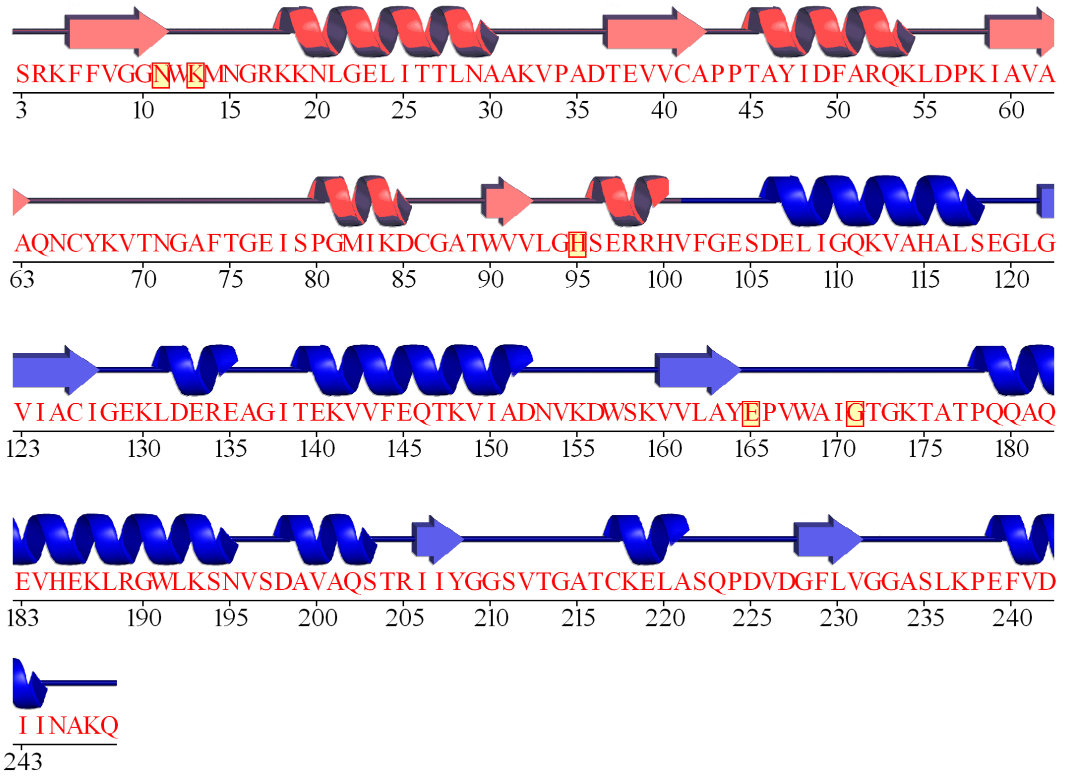

5.6. *Ribonuclease A*

Udgaonkar et al. (29) analysed the folding of bovine pancreatic ribonuclease A (PDB: 1RBX) using HDX-NMR. They identify an intermediate with the first N-terminal α-helices unformed.

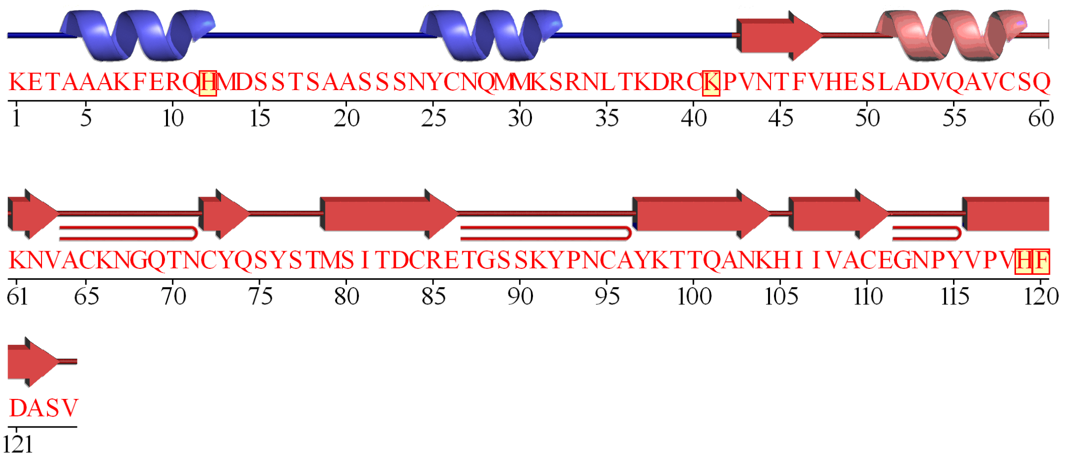

5.7. *Myoglobin*

Pan et al. (30) studied the folding of horse apo-myoglobin (PDB: 1YMB) using HDX-MS.

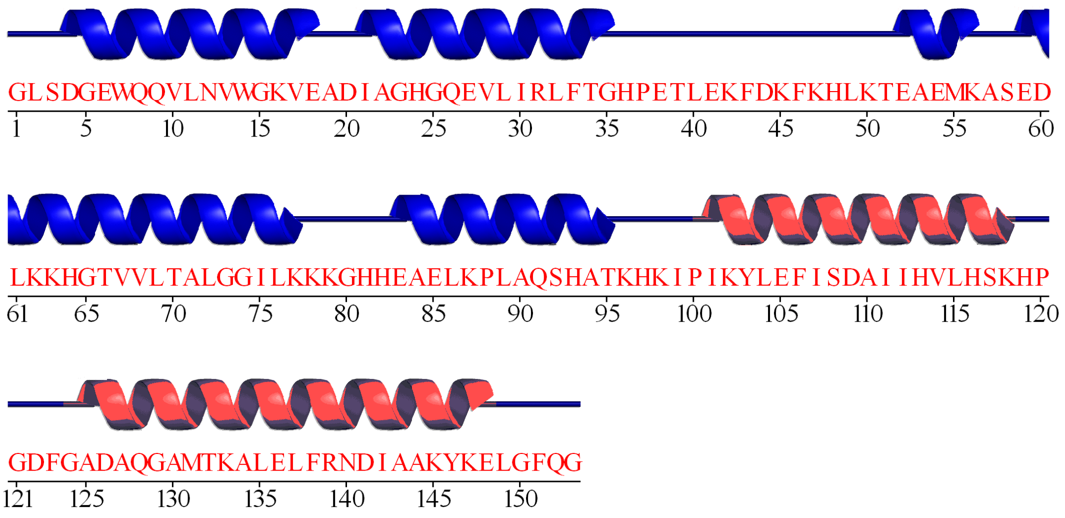

5.8. *Cardiotoxin III*

Sivaraman et al. (31) studied the folding of cardiotoxin analogue III (PDB: 2CRT), a protein present in the venom of *Naja naja atra*. This protein is a small all-β protein with two recognisable β-sheets: a double-stranded β-sheet closer to the C-terminus, and a triple-stranded β-strand closer to the N-terminus. Experimental results show that the triple-stranded β-sheet folds about 10 ms faster than the double-stranded element.

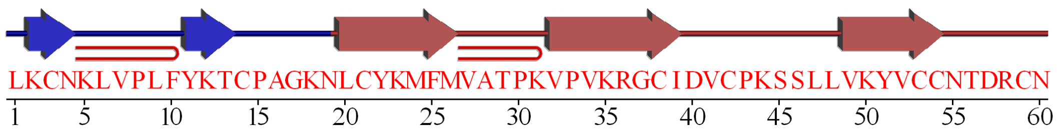

5.9. *Flavodoxin-2*

Nabuurs et al. (32) studied the folding of flavodoxin 2 (PDB: 1YOB) from *A. vinelandii* using HDX-NMR. The authors identify an off-pathway intermediate where most of the secondary structure is formed, except for two regions that adopt a random coil conformation.

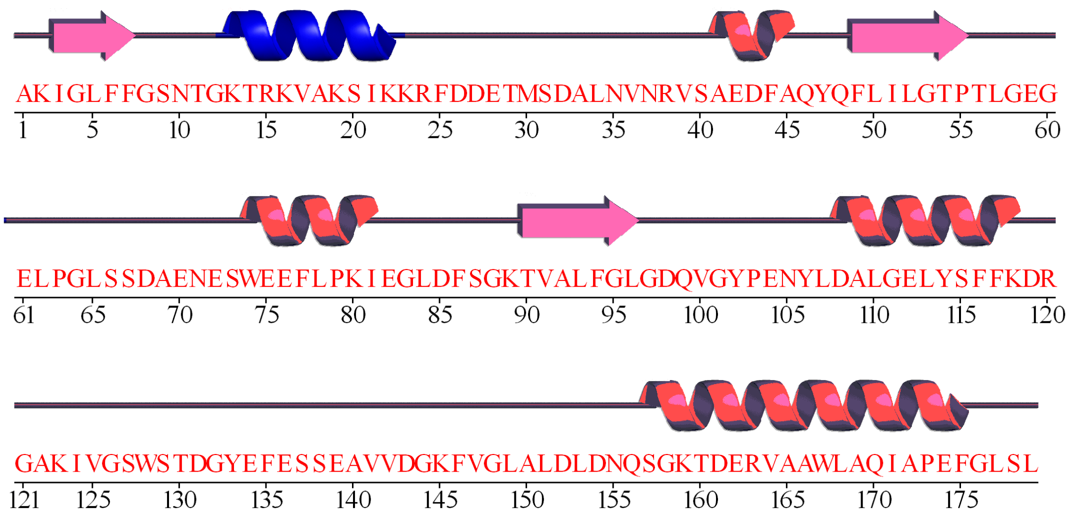
AKIGLFFGSNTGKTRKVAKSIKKRFDDETMSDALNVNRVSAEDFAQYQFLILGTPTLGEG
1 5 10 15 20 25 30 35 40 45 50 55 60
ELPGLSSDAENESWEEFLPKIEGLDFSGKTVALFGLGDQVGYPENYLDALGELYSFFKDR
61 65 70 75 80 85 90 95 100 105 110 115 120
GAKIVGSWSTDGYEFESSEAVVDGKFVGLALDLDNQSGKTDERVAAWLAQIAPEFGLSL
121 125 130 135 140 145 150 155 160 165 170 175

6. COARSE-GRAINED POTENTIAL

We employed the following native-centric potential:

$$\begin{aligned} E = & \sum_i k_b (r_i - r_0)^2 + \sum_i \sum_{j=1}^{4} k_{\phi,ij}(1+\cos(j\phi_i - \delta_{ij})) \\ & + \sum_i -\frac{1}{\gamma} \ln \left[\exp(-\gamma(k_\alpha(\theta_i - \theta_\alpha)^2 + \epsilon_\alpha) + \exp(-\gamma k_\beta(\theta_i - \theta_\beta)^2 \right] \\ & + \sum_{ij} \frac{q_i q_j e^2}{4\pi\epsilon_0\epsilon_r r_{ij}} \exp\left[-\frac{r_{ij}}{l_D}\right] + \sum_{ij\in \mathrm{NC}} \epsilon_{ij}^{\mathrm{NC}} \left[13\left(\frac{\sigma_{ij}}{r_{ij}}\right)^{12} - 18\left(\frac{\sigma_{ij}}{r_{ij}}\right)^{10} + 4\left(\frac{\sigma_{ij}}{r_{ij}}\right)^{6} \right] \\ & + \sum_{ij\in \mathrm{NN}} \epsilon_{ij}^{\mathrm{NN}} \left[13\left(\frac{\sigma_{ij}}{r_{ij}}\right)^{12} - 18\left(\frac{\sigma_{ij}}{r_{ij}}\right)^{10} + 4\left(\frac{\sigma_{ij}}{r_{ij}}\right)^{6} \right] \end{aligned} \tag{1}$$

These terms are, in order, the contributions from bonds, dihedral angles (33), bond angles (34), electrostatics, native and non-native interactions (35). The bond term is a simple harmonic potential, where k_b is the force constant, which is assigned to $100\mathrm{kJ}/(\AA \cdot \mathrm{mol})$, r_0 is the equilibrium bond length and r_i is the actual distance between the beads. The dihedral angle term is the Karanicolas-Brooks potential (33), which corresponds to a standard periodic torsion potential where the force constants $k_{\phi,ij}$, and the phases ϕ_{ij} are determined only by the second and third residue in every group of four residues defining a dihedral. The bond angle term is the Best-Hummer-Cheng potential (34), where θ_i is the bond angle, and the parameters γ, α, k_α, k_β and ϵ_α are constants (see (34) for more details).

The electrostatics term is a simple screened Coulombic potential with the charges defined by the net charge of the residues at pH=7 (*i.e.* lysine and arginine have a positive +1 charge, aspartate and glutamate have a negative -1 charge and the other residues are neutral). Finally, in the native and non-native potential, the value of $\epsilon_{ij}^{\mathrm{NC}}$, which sets the depth of the energy minimum for a native contact, is calculated as $\epsilon_{ij}^{\mathrm{NC}} = n_{ij}\epsilon_{\mathrm{HB}} + \eta\epsilon_{ij}$. Here, ϵ_{HB}, and ϵ_{ij} represent energy contributions arising from hydrogen bonding and van der Waals contacts between residues i and j identified from the all-atom structure of the protein, respectively. n_{ij} is the number of hydrogen bonds formed between residues i and j and $\epsilon_{\mathrm{HB}} = 0.75$ kcal/mol. The value of ϵ_{ij} is set on the basis of the Betancourt-Thirumalai pairwise potential (36).

7. ADDITIONAL FIGURES

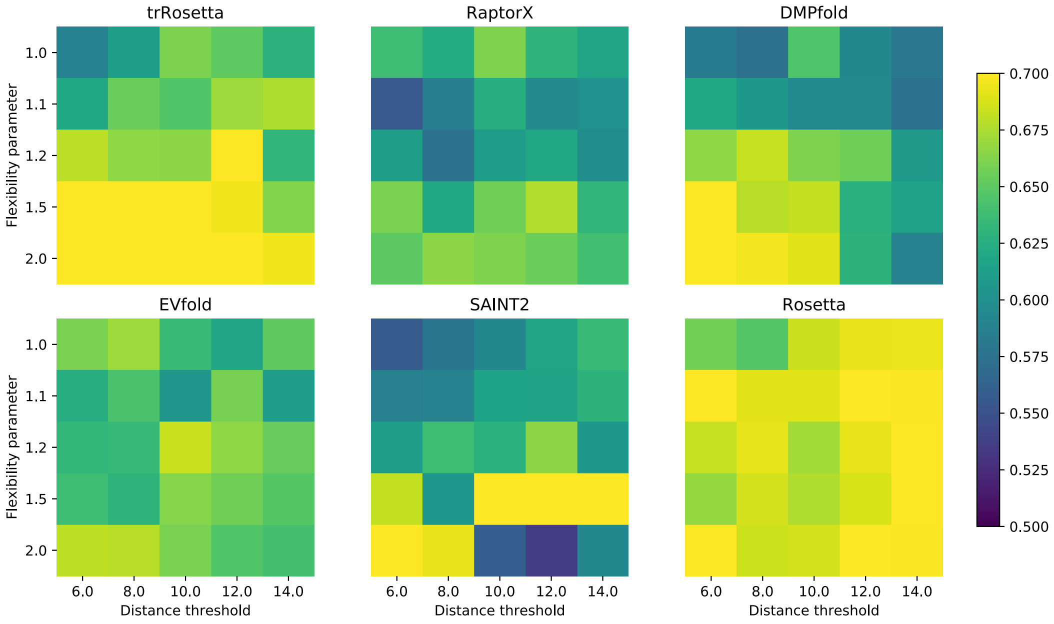

Figure S1. Parameter stability in trajectory analysis. We compare the area under the receiver-operating curve (AUROC) for the mechanism classification problem (determining if a protein folds via a two-state or multistate mechanism) for several choices of the distance threshold and flexibility hyperparameters. These results are produced using ten decoys per program for each of the 170 proteins presented in Appendix 3. These numbers suggest that different parameter choices perform better for different programs, but there is little difference overall.

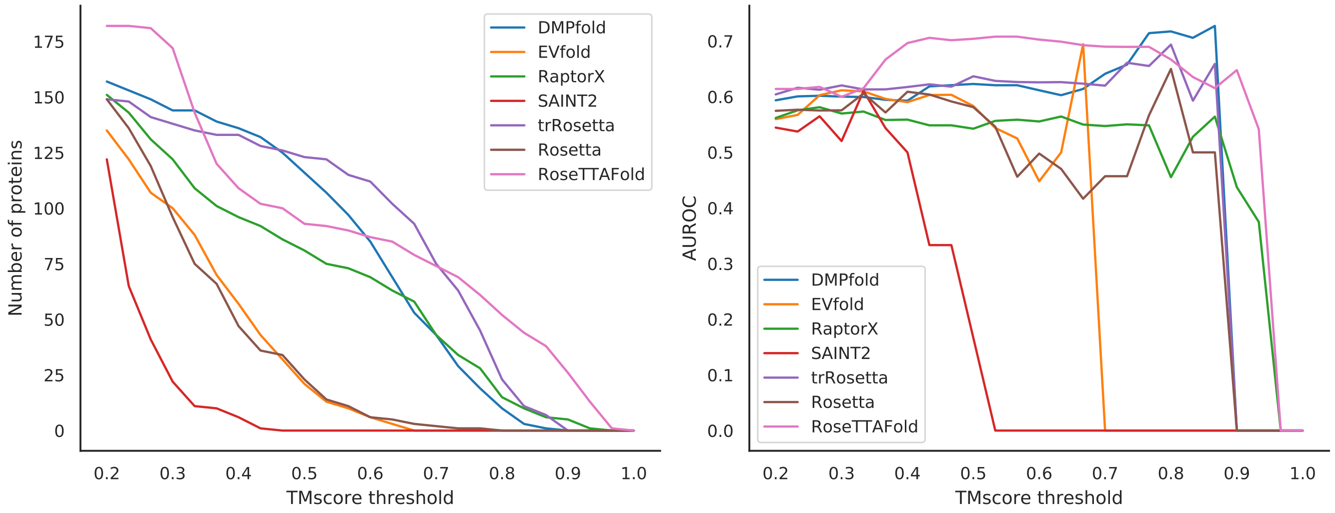

Figure S2. Predictive performance of the seven protein structure prediction programs. We compute the average TMscore (37) of the first ten decoys for each code, and use it as a proxy for the predictive performance of the algorithms. Left: cumulative number of proteins (y-axis) that were predicted with an average TMscore greater than the threshold (x-axis). The area under this curve can be interpreted as the global efficacy of the predictor. Right: area under the receiver-operator curve (y-axis) for all proteins above a given threshold (x-axis). For most proteins, the ability of simulated trajectories to distinguish formal folding kinetics is approximately independent from the predictive performance of the algorithm.

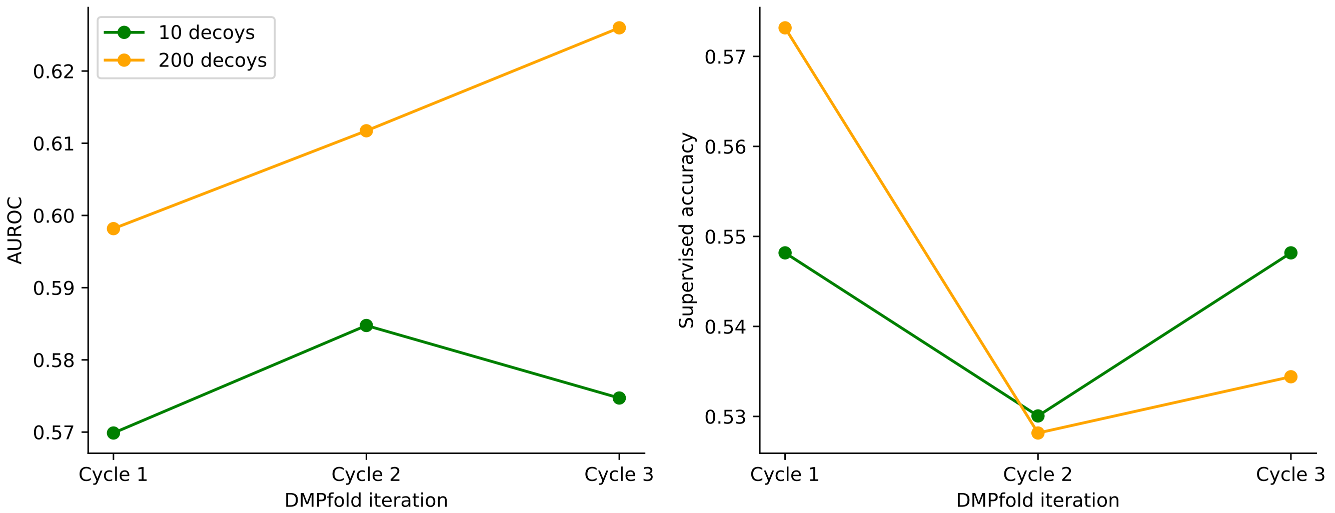

Figure S3. Change in predictive power across the different DMPfold cycles. While the reliability of the score, measured as the area under the receiver-operating curve (AUROC), seems to increase with successive cycles, the accuracy of the prediction does not improve.

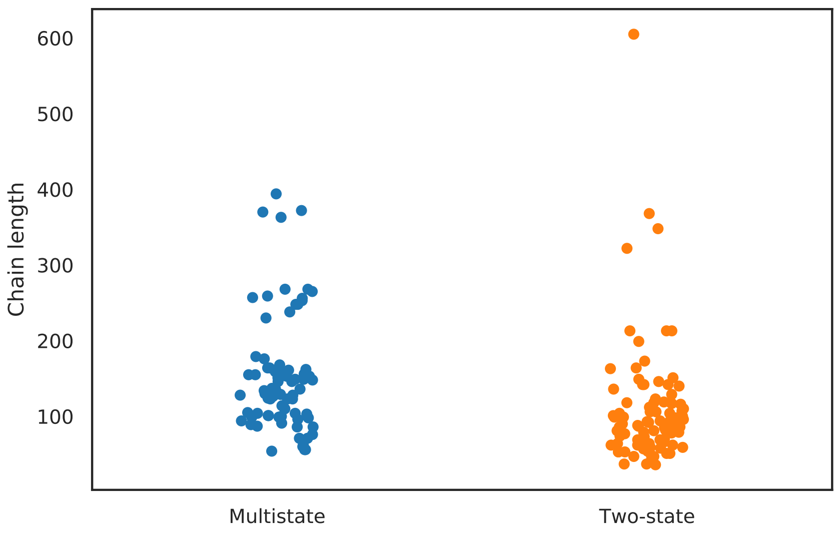

Figure S4. Distribution of lengths at each of the formal kinetics classes. There is not a trivial threshold that separates the classes.

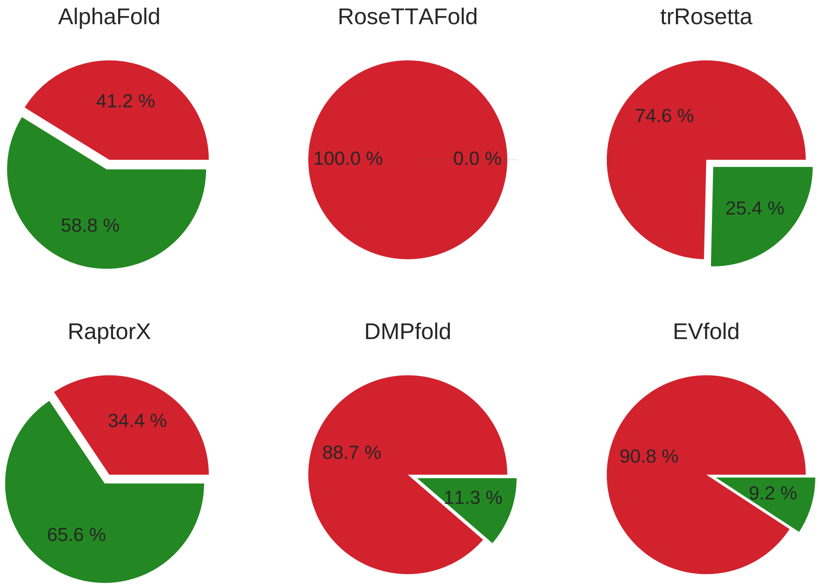

Figure S5. Mean proportion of the trajectories exhibiting significant clashes. A snapshot is considered to exhibit significant clashes if its clashscore (38) is higher than the 99th percentile for all PDB structures with resolution $\leq 2.5\AA$ (30 in the snapshot downloaded on the 2nd of July of 2021).

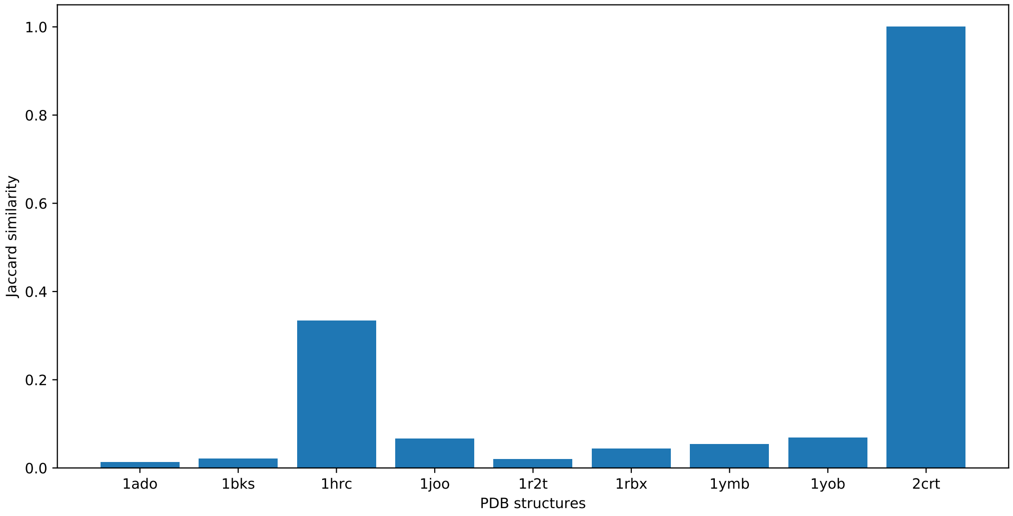

Figure S6. Jaccard similarity between the intermediates predicted by AlphaFold 2 and the ground truth. The assignments have been expressed as a binary string (where 1 means that the native contacts between secondary structure elements are formed in the intermediate, while 0 means they are not). AlphaFold 2 achieves a high score only on cardiotoxin analogue III (PDB: 2CRT), a small protein.

REFERENCES

[1]Daniel A Nissley, Ajeet K Sharma, Nabeel Ahmed, Ulrike A Friedrich, Günter Kramer, Bernd Bukau, and Edward P O'Brien. Accurate prediction of cellular co-translational folding indicates proteins can switch from post-to co-translational folding. *Nature communications*, 7(1):1–13, 2016.

[2]Daniel A Nissley and Edward P O'Brien. Structural origins of fret-observed nascent chain compaction on the ribosome. *The Journal of Physical Chemistry B*, 122(43):9927–9937, 2018.

[3]Daniel A Nissley, Quyen V Vu, Fabio Trovato, Nabeel Ahmed, Yang Jiang, Mai Suan Li, and Edward P O'Brien. Electrostatic interactions govern extreme nascent protein ejection times from ribosomes and can delay ribosome recycling. *Journal of the American Chemical Society*, 142(13):6103–6110, 2020.

[4]Shankar Kumar, John M Rosenberg, Djamal Bouzida, Robert H Swendsen, and Peter A Kollman. The weighted histogram analysis method for free-energy calculations on biomolecules. i. the method. *Journal of computational chemistry*, 13(8):1011–1021, 1992.

[5]Balachandran Manavalan, Kunihiro Kuwajima, and Jooyoung Lee. Pfdb: A standardized protein folding database with temperature correction. *Scientific reports*, 9(1):1–9, 2019.

[6]Peter Eastman, Jason Swails, John D Chodera, Robert T McGibbon, Yutong Zhao, Kyle A Beauchamp, Lee-Ping Wang, Andrew C Simmonett, Matthew P Harrigan, Chaya D Stern, et al. Openmm 7: Rapid development of high performance algorithms for molecular dynamics. *PLoS computational biology*, 13(7):e1005659, 2017.

[7]Piotr Rotkiewicz and Jeffrey Skolnick. Fast procedure for reconstruction of full-atom protein models from reduced representations. *Journal of computational chemistry*, 29(9):1460–1465, 2008.

[8]Dominik Gront, Daniel W Kulp, Robert M Vernon, Charlie EM Strauss, and David Baker. Generalized fragment picking in rosetta: design, protocols and applications. *PloS one*, 6(8):e23294, 2011.

[9]Saulo HP de Oliveira, Jiye Shi, and Charlotte M Deane. Building a better fragment library for de novo protein structure prediction. *PloS one*, 10(4):e0123998, 2015.

[10]John Jumper. Highly accurate protein structure prediction with alphafold. *Nature*.

[11]Peter S Kim and Robert L Baldwin. Specific intermediates in the folding reactions of small proteins and the mechanism of protein folding. *Annual review of biochemistry*, 51(1):459–489, 1982.

[12]Peter S Kim and Robert L Baldwin. Intermediates in the folding reactions of small proteins. *Annual review of biochemistry*, 59(1):631–660, 1990.

[13]Keith D Wilkinson. The discovery of ubiquitin-dependent proteolysis. *Proceedings of the National Academy of Sciences*, 102(43):15280–15282, 2005.

[14]Sophie E Jackson. Ubiquitin: a small protein folding paradigm. *Organic & biomolecular chemistry*, 4(10):1845–1853, 2006.

[15]Jon M Sorenson and Teresa Head-Gordon. Toward minimalist models of larger proteins: A ubiquitin-like protein. *Proteins: Structure, Function, and Bioinformatics*, 46(4):368–379, 2002.

[16]Neelan J Marianayagam and Sophie E Jackson. The folding pathway of ubiquitin from all-atom molecular dynamics simulations. *Biophysical chemistry*, 111(2):159–171, 2004.

[17]Stefano Piana, Kresten Lindorff-Larsen, and David E Shaw. Atomic-level description of ubiquitin folding. *Proceedings of the National Academy of Sciences*, 110(15):5915–5920, 2013.

[18]Sepideh Khorasanizadeh, Iain D Peters, Tauseef R Butt, and Heinrich Roder. Folding and stability of a tryptophan-containing mutant of ubiquitin. *Biochemistry*, 32(27):7054–7063, 1993.

[19]Bryan A Krantz and Tobin R Sosnick. Distinguishing between two-state and three-state models for ubiquitin folding. *Biochemistry*, 39(38):11696–11701, 2000.

[20]Heather M Went, Claudia G Benitez-Cardoza, and Sophie E Jackson. Is an intermediate state populated on the folding pathway of ubiquitin? *FEBS letters*, 567(2-3):333–338, 2004.

[21]Sebastian Meier, Mark Strohmeier, Martin Blackledge, and Stephan Grzesiek. Direct observation of dipolar couplings and hydrogen bonds across a β-hairpin in 8 m urea. *Journal of the American Chemical Society*, 129(4):754–755, 2007.

[22]Hai Pan and David L Smith. Quaternary structure of aldolase leads to differences in its folding and unfolding intermediates. *Biochemistry*, 42(19):5713–5721, 2003.

[23]Patrick L Wintrode, Teerapat Rojsajjakul, Ramakrishna Vadrevu, C Robert Matthews, and David L Smith. An obligatory intermediate controls the folding of the α-subunit of tryptophan synthase, a tim barrel protein. *Journal of molecular biology*, 347(5):911–919, 2005.

[24]Heinrich Roder, Gülnur A Elöve, and S Walter Englander. Structural characterization of folding intermediates in cytochrome c by h-exchange labelling and proton nmr. *Nature*, 335(6192):700–704, 1988.

[25]Gulnur A Elove, Abani K Bhuyan, and Heinrich Roder. Kinetic mechanism of cytochrome c folding: involvement of the heme and its ligands. *Biochemistry*, 33(22):6925–6935, 1994.

[26]Hossein Fazelinia, Ming Xu, Hong Cheng, and Heinrich Roder. Ultrafast hydrogen exchange reveals specific structural events during the initial stages of folding of cytochrome c. *Journal of the American Chemical Society*, 136(2):733–740, 2014.

[27]William F Walkenhorst, Jason A Edwards, John L Markley, and Heinrich Roder. Early formation of a beta hairpin during folding of staphylococcal nuclease h124l as detected by pulsed hydrogen exchange. *Protein science*, 11(1):82–91, 2002.

[28]Hai Pan, Ashraf S Raza, and David L Smith. Equilibrium and kinetic folding of rabbit muscle triosephosphate isomerase by hydrogen exchange mass spectrometry. *Journal of molecular biology*, 336(5):1251–1263, 2004.

[29]Jayant B Udgaonkar and Robert L Baldwin. Early folding intermediate of ribonuclease a. *Proceedings of the National Academy of Sciences*, 87(21):8197–8201, 1990.

[30]Jingxi Pan, Jun Han, Christoph H Borchers, and Lars Konermann. Characterizing short-lived protein folding intermediates by top-down hydrogen exchange mass spectrometry. *Analytical chemistry*, 82(20):8591–8597, 2010.

[31]Thirunavukkarasu Sivaraman, Thallampuranam Krishnaswamy S Kumar, Ding Kwo Chang, Wann Yin Lin, and Chin Yu. Events in the kinetic folding pathway of a small, all β-sheet protein. *Journal of Biological Chemistry*, 273(17):10181–10189, 1998.

[32]Sanne M Nabuurs, Adrie H Westphal, and Carlo PM van Mierlo. Noncooperative formation of the off-pathway molten globule during folding of the α- β parallel protein apoflavodoxin. *Journal of the American Chemical Society*, 131(7):2739–2746, 2009.

[33]John Karanicolas and Charles L Brooks III. The origins of asymmetry in the folding transition states of protein l and protein g. *Protein Science*, 11(10):2351–2361, 2002.

[34]Robert B Best, Yng-Gwei Chen, and Gerhard Hummer. Slow protein conformational dynamics from multiple experimental structures: the helix/sheet transition of arc repressor. *Structure*, 13(12):1755–1763, 2005.

[35]Edward P O'Brien, John Christodoulou, Michele Vendruscolo, and Christopher M Dobson. Trigger factor slows co-translational folding through kinetic trapping while sterically protecting the nascent chain from aberrant cytosolic interactions. *Journal of the American Chemical Society*, 134(26):10920–10932, 2012.

[36]Marcos R Betancourt and D Thirumalai. Pair potentials for protein folding: choice of reference states and sensitivity of predicted native states to variations in the interaction schemes. *Protein science*, 8(2):361–369, 1999.

[37]Yang Zhang and Jeffrey Skolnick. Tm-align: a protein structure alignment algorithm based on the tm-score. *Nucleic acids research*, 33(7):2302–2309, 2005.

[38]Ian W Davis, Laura Weston Murray, Jane S Richardson, and David C Richardson. Molprobity: structure validation and all-atom contact analysis for nucleic acids and their complexes. *Nucleic acids research*, 32(suppl_2):W615–W619, 2004.
